# Inhibition of histone acetyltransferase GCN5 extends lifespan in both yeast and human cell lines

**DOI:** 10.1111/acel.13129

**Published:** 2020-03-11

**Authors:** Boyue Huang, Dandan Zhong, Jie Zhu, Yongpan An, Miaomiao Gao, Shuai Zhu, Weiwei Dang, Xin Wang, Baoxue Yang, Zhengwei Xie

**Affiliations:** ^1^ State Key Laboratory of Natural and Biomimetic Drugs Department of Pharmacology School of Basic Medical Sciences Peking University Beijing China; ^2^ Huffington Center on Aging Baylor College of Medicine Houston TX USA; ^3^ Key Laboratory of Molecular Cardiovascular Sciences Ministry of Education Beijing China; ^4^ Peking University International Cancer Institute Peking University Beijing China

**Keywords:** anti‐aging, GCN5, histone acetyltransferases, microfluidics, NGG1

## Abstract

Histone acetyltransferases (HATs) are important enzymes that transfer acetyl groups onto histones and thereby regulate both gene expression and chromosomal structures. Previous work has shown that the activation of sirtuins, which are histone deacetylases, can extend lifespan. This suggests that inhibiting HATs may have a similar beneficial effect. In the present study, we utilized a range of HAT inhibitors or heterozygous Gcn5 and Ngg1 mutants to demonstrate marked yeast life extension. In human cell lines, HAT inhibitors and selective RNAi‐mediated Gcn5 or Ngg1 knockdown reduced the levels of aging markers and promoted proliferation in senescent cells. Furthermore, this observed lifespan extension was associated with the acetylation of histone H3 rather than that of H4. Specifically, it was dependent upon H3K9Ac and H3K18Ac modifications. We also found that the ability of caloric restriction to prolong lifespan is Gcn5‐, Ngg1‐, H3K9‐, and H3K18‐dependent. Transcriptome analysis revealed that these changes were similar to those associated with heat shock and were inversely correlated with the gene expression profiles of aged yeast and aged worms. Through a bioinformatic analysis, we also found that HAT inhibition activated subtelomeric genes in human cell lines. Together, our results suggest that inhibiting the HAT Gcn5 may be an effective means of increasing longevity.

## Graphical abstract

The partial inhibition of Gcn5 extends lifespan. Such extension, as well as Caloric restriction, depends on HAT Gcn5, the linker protein Ngg1, H3K9Ac, and H3K18Ac. Histone acetyltransferase inhibition reversed the transcriptome in aged yeast and activated subtelomeric genes in human cells, inducing downstream stress response genes.

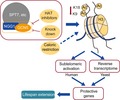

## 1 INTRODUCTION

Aging is a process in which an organism suffers from a rising risk of death accompanied by a progressive decline in functional and physiological integrity (Lopez‐Otin, Blasco, Partridge, Serrano, & Kroemer, 2013). At present, known interventions reliably slowing the aging process in humans include exercise and caloric restriction (Fontana & Klein, 2007). Epigenetic modification is one of the key hallmarks of aging and can result in genome‐wide changes in chromosomal structure and gene expression, thereby broadly impacting key cellular processes (Benayoun, Pollina, & Brunet, 2015).

One common form of epigenetic modification is the acetylation of histones, and particularly of the H3 and H4 histones, leading to an increase in the transcription of proximal genes (Eberharter & Becker, 2002). Histone acetyltransferases (HATs) are the enzymes which mediate the acetylation of these proteins, whereas histone deacetylases (HDACs) oppose this process. To mediate histone acetylation, HATs transfer an acetyl‐CoA‐derived acetyl group onto the lysine residues of target histones. In previous aging studies, the majority of epigenetic interest has focused on a family of HDACs known as sirtuins. Studies have suggested that the HDAC inhibitor resveratrol can extend lifespan, but this effect was not detectable in studies in mice (Pearson et al., 2008). As such, it remains uncertain which HDACs/HATs regulate longevity, and thus, more efforts need to be made to identify targets that can increase the lifespan of complex organisms.

Histone acetyltransferases regulate a wide range of processes in the cells in which they act owing to their ability to activate target gene transcription (Kurdistani & Grunstein, 2003). Specific HAT complexes, such as GCN5‐related N‐acetyltransferases (GNATs) and MYST‐related HATs, are known to exist, but their selectivity in the regulation histone acetylation remains uncertain (Brown, Lechner, Howe, & Workman, 2000). Gcn5 was the first HAT identified which was known to be linked to transcription, and as such, it has been the study of extensive research (Brownell et al., 1996). GCN5 has been found to be essential for the retention of circular DNA within mother cells, which causes the accumulation of nuclear pore complexes and affects the organization of the aging nuclei, and as such, it was hypothesized to be important for the aging process (Denoth‐Lippuner, Krzyzanowski, Stober, & Barral, 2014). In yeast that lack the genes SGF73, SGF11, and UBP8, which encode the SAGA/SLIK complex histone deubiquitinase module, lifespan is known to be increased in a manner dependent upon GCN5 (Jiang et al., 2016). Deleting the *gcn5* gene alone, however, does not increase the lifespan of cells, and homozygous knockout of *gcn5* causes murine embryos to be malformed by E8.5 and to die by E11 (Lin et al., 2008), suggesting GCN5 is essential for developmental processes. Gcn5 is known to mediate H3 acetylation at both lysine 9 and 18. The physiological importance of these specific site preferences in the context of aging, however, remains uncertain and requires further study.

In this study, we offer novel evidence indicating that the partial inhibition of specific HATs can mediate the rejuvenation of yeast and human cell lines. This increase in lifespan is achieved via disrupting H3 acetylation that is dependent upon Gcn5 and the linked protein Ngg1. Using site‐specific mutations, we were able to confirm that Gcn5 preferentially mediates H3 acetylation on K9 and K18 residues and that acetylation of these two sites is associated with the observed lifespan extension. We also used low glucose media in order to demonstrate the ability of HAT inhibition to mimic the effects of caloric restriction. Through RNA sequencing, we further determined that HAT inhibitors largely influenced the expression of genes found in subtelomeric domains. In knockdown cell lines, we observed both delayed replicative senescence and decreased markers of aging.

## 2 RESULTS

### 2.1 HAT inhibition increases the lifespan of yeast and human cell lines

**Figure 1:**
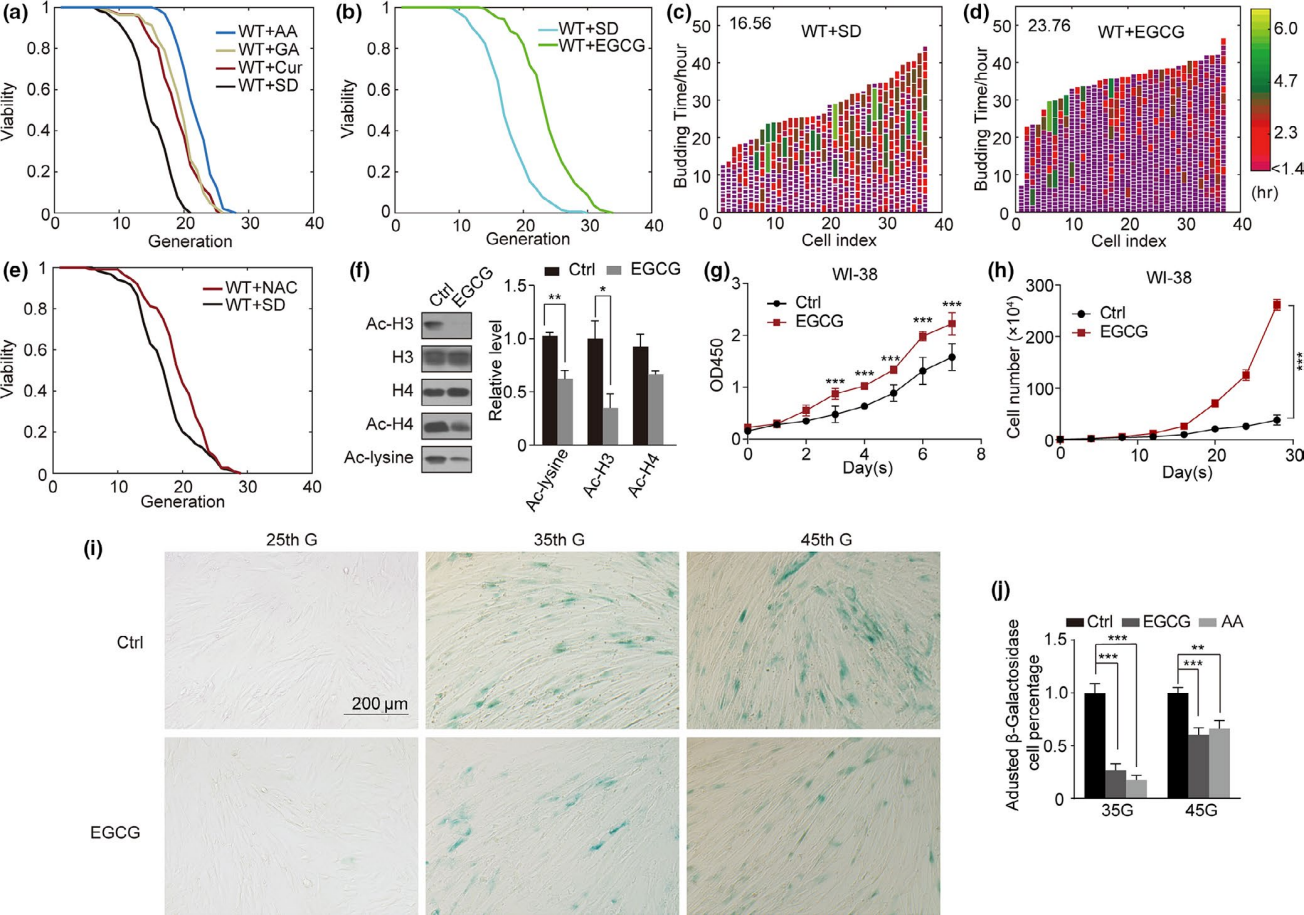
HAT inhibitors increased the lifespan of both yeast and human cell lines. (a) HAT inhibitors increased the replicative lifespan of treated cells as compared to wild‐type (WT) cells in SD media. (b) EGCG increased WT cell lifespan. (c–d) Budding profiles of mother cells for (c) WT + SD, (d) WT + EGCG, exhibiting the duration and heterogeneity of the cell cycle (note that the color scale is exponential; cycles 1.4 hr or less in duration were purple). (e) NAC effects on cell lifespan. (f) Ac‐lysine, Ac‐H3, H3, H4, and Ac‐H4 levels for cells grown in SD and EGCG media as measured via Western blotting (*n* = 3). (g) WI‐38 cells stained for SA‐β‐gal for three periods following 10 µM EGCG or DMSO vehicle treatment. (h) Measurement of senescence‐associated β‐galactosidase activity in WI‐38 cells following 10 µM EGCG, 2.5 µM AA, or DMSO vehicle treatment. (i) EGCG effects on WI‐38 cell division over a 1‐week period. (j) Comparison of EGCG and DMSO vehicle control effects on WI‐38 cell growth over a 1‐month period

As the activation of Sir2 has been linked to the prolongation of model organism lifespans (Imai, Armstrong, Kaeberlein, & Guarente, 2000), we wanted to assess whether inhibiting HATs would achieve a similar effect. We employed two types of microfluidic chips in this study. One is the island chip derived from a previous study (Zhang et al., 2012). The other is a modified U‐shape chip (Jo, Liu, Gu, Dang, & Qin, 2015), as shown in Figure S1a. Cells were pumped in using a microfluidic device, and the budding timing across the entirety of the lifespan was continuously monitored for 60h via repeated microscopic imaging. We found that the HAT inhibitors epigallocatechin gallate (EGCG)(Choi et al., 2009), anacardic acid (AA), garcinol (GA), and curcumin all prolonged the replicative lifespan of these cells by >50%, 50%, 33%, and 29%, respectively (Figure 1a,b). Cell cycle length throughout the entire yeast lifespan was correspondingly reduced, with cells dividing more smoothly following HAT inhibition (Figure 1c,d, Figure S1b–d). It is important to note that HAT inhibitors not only extend lifespan but also prevent cell cycle extension at the end of life. In yeast, this corresponds to a suppression of the reduction in health at the end of life. As EGCG achieved the most marked lifespan extension, it was used for further experimentation. Epigallocatechin gallate has been experimentally reported to increase the lifespan of worms, *Drosophila melanogaster* (Wagner et al., 2015), and rats (Niu et al., 2013). Previous studies have attributed such extensions to the antioxidant activity of EGCG. As such, we assessed the ability of the strong antioxidant N‐acetylcysteine (NAC) (Zafarullah, Li, Sylvester, & Ahmad, 2003), to extend yeast lifespan, revealing that it only mediated a 5% increase in yeast lifespan—a less dramatic increase than that observed upon EGCG treatment (Figure 1e, Figure S1e). This suggests that other factors beyond antioxidant activity are linked to EGCG‐mediated yeast lifespan prolongation. We therefore hypothesized that it facilitates this effect via its HAT‐inhibitory activity. Indeed, we found that EGCG treatment was associated with reduced overall Ac‐lysine levels (Figure 1f), with a preferential impact on Ac‐H3 relative to Ac‐H4 expression (Figure 1f). These results indicate that HAT inhibition can mediate a substantial increase in yeast lifespan in a manner potentially linked to H3 histone acetylation.

In order to assess how HAT inhibition influences human cell lines, we next assessed the division curves of the 2BS and WI‐38 cell lines over a 1‐week period. These cells are embryonic lung fibroblasts commonly used to model aging, and we found that their replicative lifespan was extended by EGCG (Figure 1g, h) and AA (Figure S2a–f). We further performed SA‐β‐gal staining in order to assess cellular senescence, revealing that HAT inhibition significantly reduced SA‐β‐gal staining after 24 hr in WI‐38 cells (Figure 1i,j, Figure S2g). Together, these findings suggest that HAT inhibition can extend the replicative lifespan and reduce senescence in human cells, rejuvenating senescent cells to some extent.

### 2.2 Deletion of GCN5 completely abolishes the lifespan extension effects of HAT inhibition whereas GCN5 knockdown leads to lifespan extension

**Figure 2:**
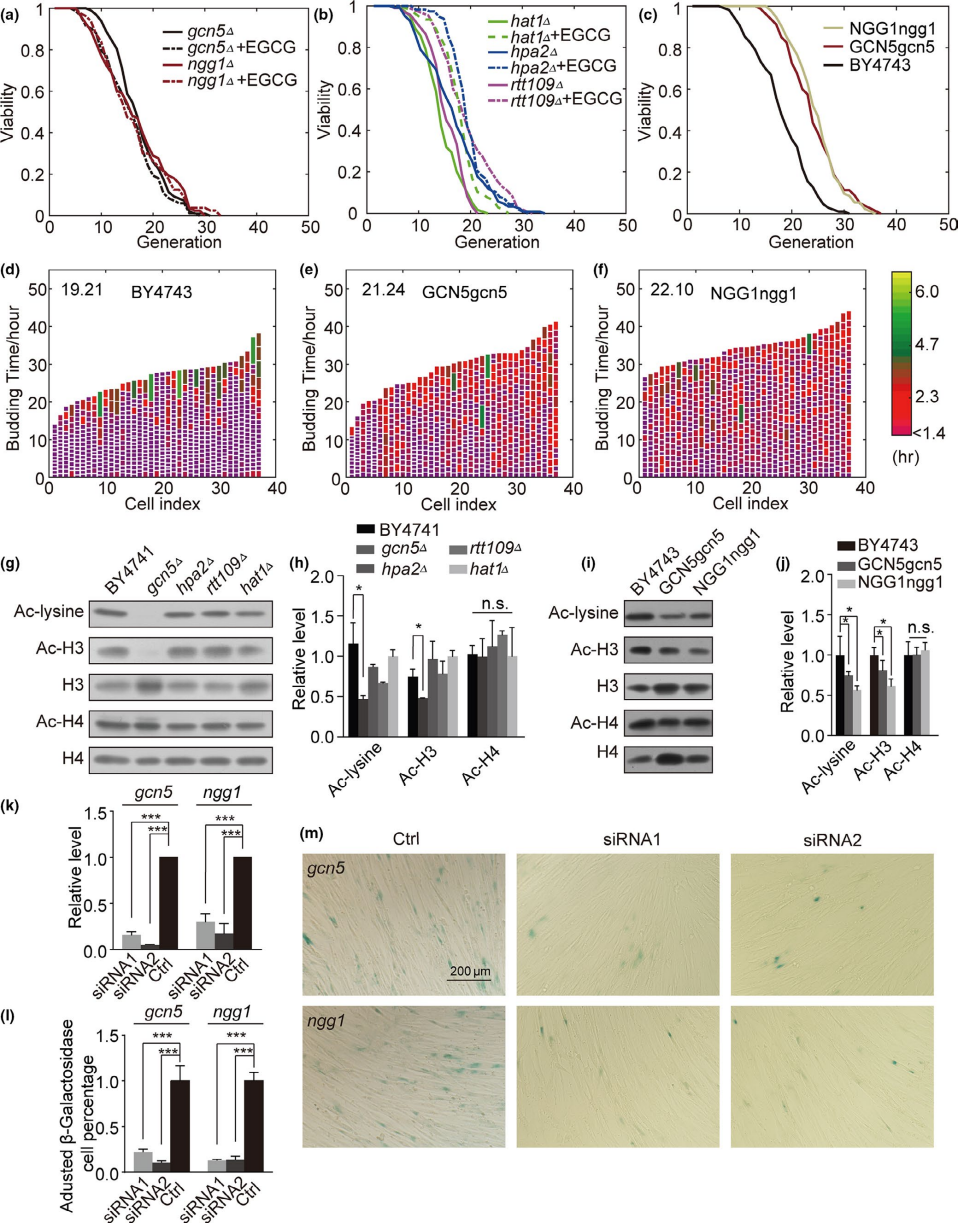
Deletion of GCN5 can abolish lifespan extension, but GCN5 knockdown rescues this phenotype. (a) EGCG effects on *gcn5Δ* and *ngg1Δ* deletion cell strains. (b) EGCG effects on HAT deletion cell strains. (c) Heterozygous diploid cell lifespans for the NGG1ngg1, GCN5gcn5, and BY4743 strains. (d–f) Budding profiles of mother cells for (d) BY4743, (e) GCN5gcn5, (f) NGG1ngg1 (exponential color scale; cell cycles of 1.4 hr or less in duration are purple; [n]: 37; average lifespan is in the upper left). (g) Ac‐lysine, Ac‐H3, H3, H4, and Ac‐H4 expression in BY4741, *gcn5Δ*, hpa2*Δ*, *rtt109Δ*, and *hat1Δ* strains as measured via Western blotting. (h) Western blotting statistics (*n* = 3). (i) Ac‐lysine, Ac‐H3, H3, Ac‐H4, and H4 expression in BY4743, GCN5gcn5 and NGG1ngg1 cells. (j) Western blotting statistics (*n* = 3). (k) Quantification of GCN5 and NGG1 expression in *gcn5* or *ngg1* knockdown WI‐38 cells. (l) Quantification of SA‐β‐gal staining. (m), SA‐β‐gal staining in *gcn5* or *ngg1* knockdown WI‐38 cells

In order to determine which HATs were associated with the observed lifespan extension effect, we used EGCG to treat cells in which key HATs including Hat1, Hpa2, Rtt109, or Gcn5 (Lee & Workman, 2007) had been knocked out. We found that EGCG still extended the lifespan of *hat1Δ, hap2Δ,* and *rtt109Δ* mutant yeast. The lifespan extension effect in the *gcn5Δ* strain*,* however, was completely absent (Figure 2a, Figure S3a–d). Levels of histone acetylation in the *hat1Δ, hap2Δ,* and *rtt109Δ* strains, characterized by Western blotting, were comparable to those in WT cells (Figure 2b, Figure S3e,f)*.* This suggests that Gcn5 is the key mediator of lifespan extension in response to EGCG. A direct measurement of the interaction between EGCG and Gcn5 was also performed. The split band in our SDS‐PAGE results (Figure S2h) indicates that EGCG and Gcn5 covalently bond to form a stable complex. To further confirm role of Gcn5 in this context, we also examined the linker protein Ngg1, which anchors Gcn5 to the SAGA/ADA complex (Horiuchi, Silverman, Marcus, & Guarente, 1995). We found that Ngg1 behaves almost identically to Gcn5 with respect to its impact on lifespan (Figure 2a).

As Gcn5 is important for the regulation of transcriptional activity across a wide range of genes, we hypothesized that while the deletion of this gene does not improve lifespan (Kim, Ohkuni, Couplan, & Jazwinski, 2004), a partial knockdown of it may do so. We therefore mated the WT and *gcn5Δ*/*ngg1Δ* strains to generate a heterozygous diploid yeast strain with a single copy of GCN5/NGG1. We found that this partial GCN5/NGG1 knockdown significantly extended lifespan relative to the control BY4743 strain (Figure 2c–f). Consistent with observations made using inhibitors, we further found that levels of H3 but not H4 acetylation were decreased in *gcn5Δ* (Figure 2g,h) and GCN5gcn5/NGG1ngg1 yeast (Figure 2i,j). Together, these findings indicate that the partial inhibition of GCN5/NGG1 was sufficient to extend yeast replicative lifespan, whereas full GCN5/NGG1 deletion eliminates this effect.

As extrachromosomal rDNA circles (ERC) are an important factor involved in yeast aging, we also tested whether the inhibition of Gcn5 activity extends replicative lifespan by impacting the stability of the rDNA tandem array. We treated *fob1∆* with EGCG, AA, and GA. We found that these treatments did not extend the lifespan of *fob1∆* further (Figure S2i), indicating that HAT inhibitors exert their effects through pathways overlapping with ERCs. It has been reported that the deletion of GCN5 suppressed the extension of replicative lifespan afforded by the induction of the retrograde response (Kim et al., 2004). This suggests that the lifespan extending effect of *fob1∆* is likely to related to GCN5, which is important for the modulation of the retrograde response and genomic stability.

In human cell lines, total GCN5 knockout can promote apoptotic cell death (Xu et al., 2000) and stably knocking this gene down in human cells is challenging. As such, we instead opted to use siRNA to achieve GCN5 knockdown (Figure 2k,l). We found that siRNA‐mediated *gcn5* and *ngg1* knockdown was able to rejuvenate senescent cells (Figure 2m), with SA‐β‐gal staining being used to quantify senescence.

### 2.3 GCN5 extends replicative lifespan in a manner dependent upon NGG1 and H3K9/H3K18 acetylation

**Figure 3:**
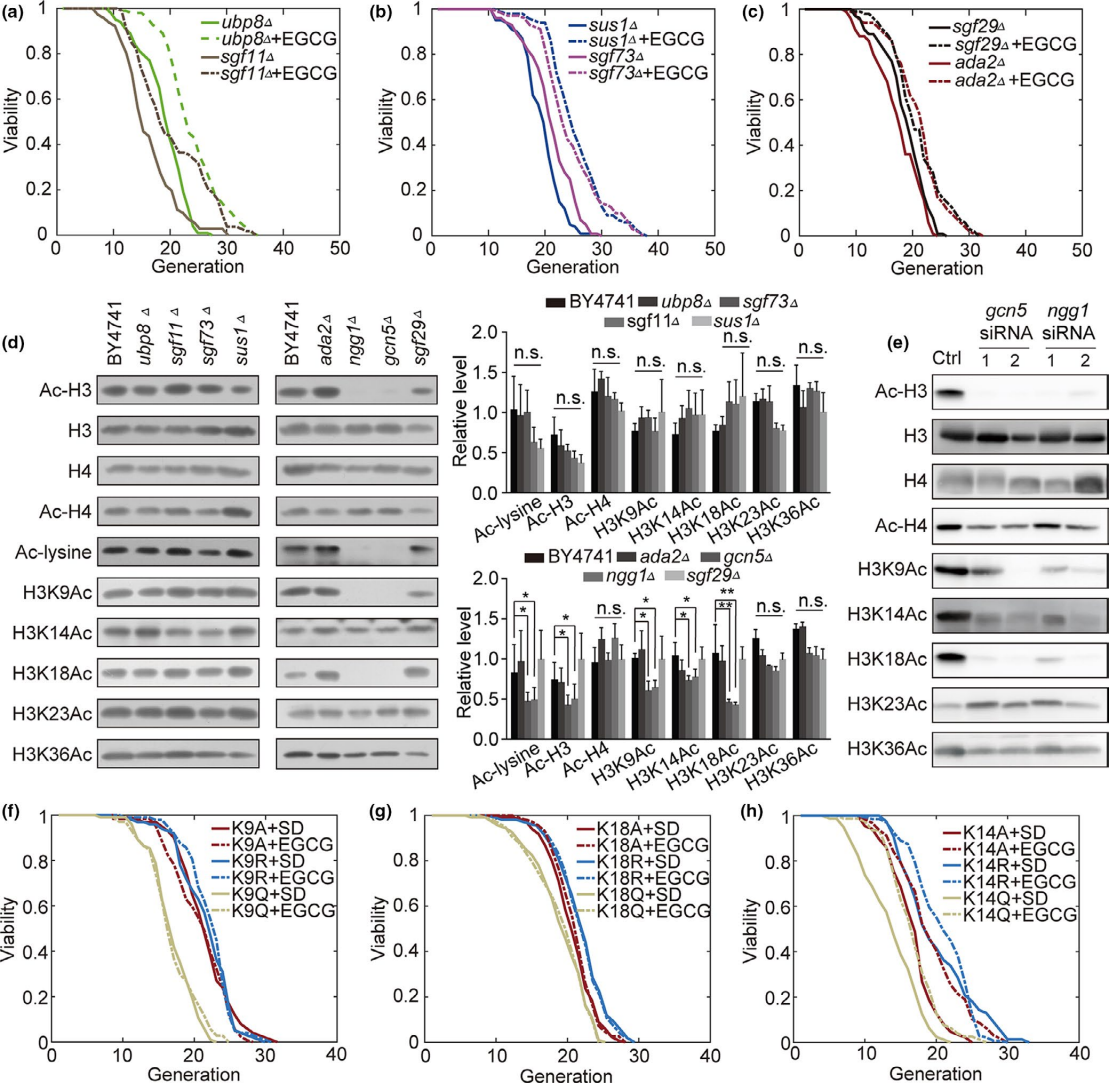
Gcn5 increases the lifespan of cells via promoting an interaction with Ngg1 and target H3K9 and H3K18 residues. (a & b) EGCG effects on SAGA subunit deletion strains. (c) EGCG effects on ADA subunit deletion strains. (d) Ac‐H3, H3, H4, Ac‐H4, Ac‐lysine, H3K9Ac, H3K14Ac, H3K18Ac, H3K23Ac, and H3K36Ac expression in different subunits of SAGA‐ and ADA‐KO cell lines as measured via Western blotting, with statistics shown (*n* = 3). (e) Ac‐H3, H3, H4, Ac‐H4, H3K9Ac, H3K14Ac, H3K18Ac, H3K23Ac, and H3K36Ac expression in control, *gcn5* knockdown, and *ngg1* knockdown WI‐38 cells measured via Western blotting. (f) K9A, K9R, and K9Q cell lifespans after growth in SD and EGCG media. (g) K18A, K18R, and K18Q lifespan following growth in SD and EGCG media. (h) K14A, K14R, and K14Q lifespan following growth in SD and EGCG media

In yeast, the ability of GCN5 to acetylate nucleosomes is dependent upon the large SAGA and ADA complexes (Armour et al., 2013). SAGA contains both Gcn5 and Ubp8, which can, respectively, acetylate and deubiquitinate residues in a controlled manner to regulate the expression of target genes (Rodriguez‐Navarro, 2009). To determine which Gcn5‐subunit interactions are important for lifespan extension, we assessed the lifespan and acetylation levels in the *sgf11Δ*, *ubp8Δ*, *sgf73Δ*, *sus1Δ*, *ngg1Δ*, *ada2Δ*, *gcn5Δ,* and *sgf29Δ* strains. We found *ubp8Δ, sgf11Δ, sgf73Δ*, *sus1Δ,* and *ada2Δ* did not disrupt the lifespan extension effect of EGCG (Figure 3a–c) and did not alter Ac‐H3 levels (Figure 3d). As such, EGCG is not dependent upon the deubiquitination function of SAGA complex.

Multiple lysine residues are located on H3. In order to determine through which of these sites Gcn5 exerts its function, we measured acetylation levels in various mutant strains and found H3K9 and H3K18 acetylation were decreased in the *gcn*5*Δ* and *ngg1Δ* strains, whereas they were unchanged in other mutants (Figure 3d). The same result was observed in yeasts given inhibitors or heterozygous diploids (Figure S3g,h). This suggested that Gcn5 and Ngg1 interact to target H3K9 and H3K18 and to thereby extend the lifespan of yeast. Together, these results suggest that there is cooperation between Gcn5 and Ngg1 that mediates selective regulation of specific H3 lysine residues in a manner that ultimately facilitates lifespan extension.

In human cell lines, siRNA‐mediated knockdown of GCN5 and NGG1 using the constructs designed above decreased Ac‐H3, H3K9Ac, and H3K18Ac levels (Figure 3e), consistent with our findings in yeast.

The disruption of H3K9 and K18 acetylation in the *gcn5Δ* or *ngg1Δ* strains shown above suggests that one or both of these modifications may have a causal relationship with lifespan extension. To test this directly, we used yeast strains in which H3K18 is modified to arginine (K18R) or glutamine (K18Q) to mimic the nonacetylated or acetylated state, respectively, demonstrating that all forms of this mutation abolished EGCG‐mediated lifespan extension (Figure 3f,g). The same was true for H3K9A, H3K9R, H3K9Q, H3K18A, H3K18R, and H3K18Q mutants (Figure 3f,g). In contrast, in H3K14A, H3K14R, and H3K14Q mutants, EGCG still mediated lifespan extension (Figure 3h). Together, these results suggest that both H3K9 and H3K18 acetylation are key targets of *Gcn5* and *Ngg1*, thereby controlling the replicative lifespan of yeast.

### 2.4 Caloric restriction‐mediated lifespan extension is dependent upon acetyltransferase activity

**Figure 4:**
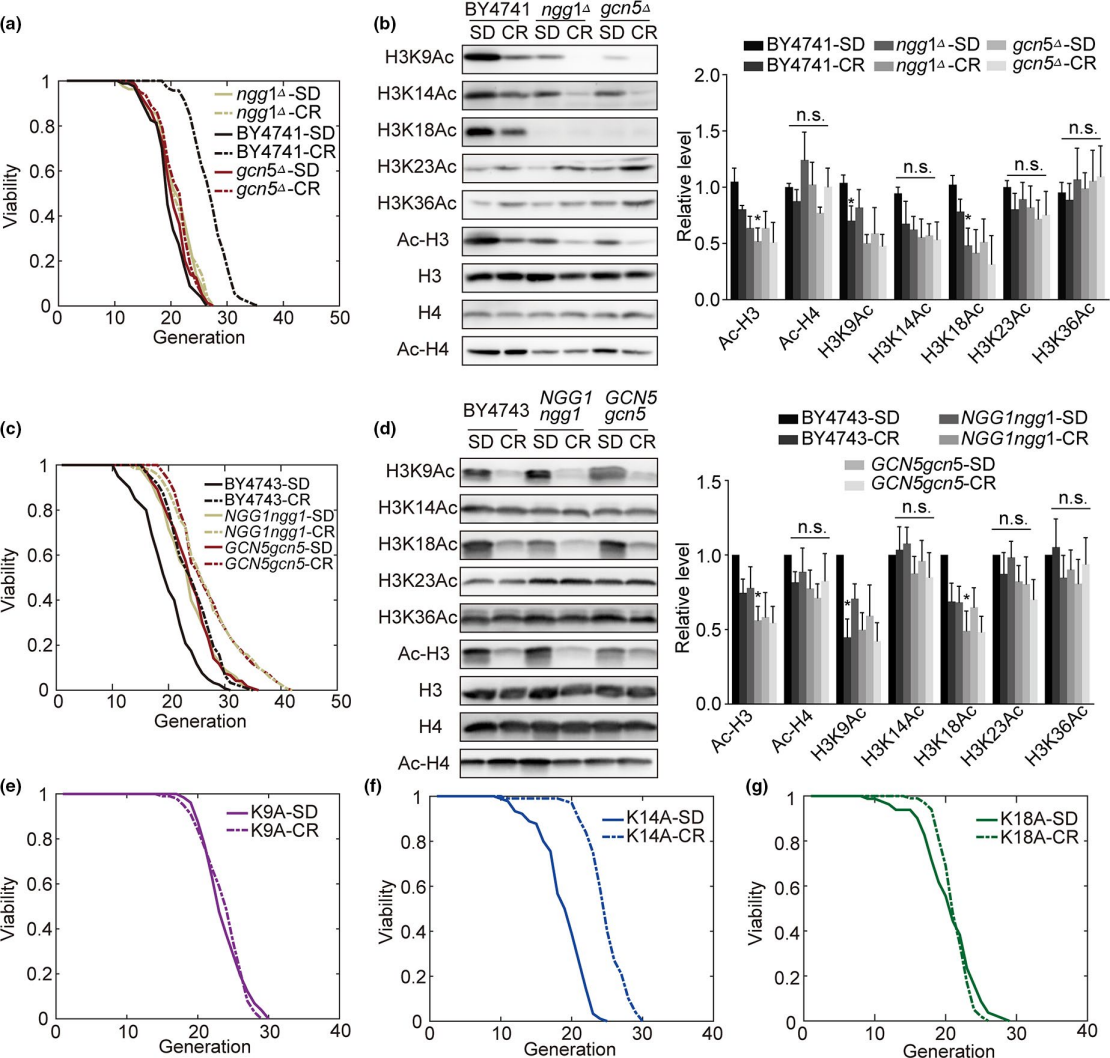
Caloric restriction increases lifespan in a manner that is dependent upon acetyltransferase activity. (a) *ngg1Δ*, BY4741 and *gcn5Δ* cell lifespans following growth using SD and low glucose medium. (b) H3K9Ac, H3K14Ac, H3K18Ac, H3K23Ac, H3K36Ac, Ac‐H3, H3, H4, and Ac‐H4 expression in BY4741, *gcn5Δ,* and *ngg1Δ* cells grown with SD and low glucose media as assessed by Western blotting (*n* = 4). (c) BY4743, NGG1ngg1, and GCN5gcn5 cell lifespans following growth using SD and low glucose medium. (d) H3K9Ac, H3K14Ac, H3K18Ac, H3K23Ac, H3K36Ac, Ac‐H3, H3, H4, and Ac‐H4 expression in BY4743, NGG1ngg1, and GCN5gcn5 cells grown with SD and low glucose media as assessed by Western blotting (*n* = 6). (e–g) Lifespan of H3K9A (e), H3K14A (f), and H3K18A (g) cells grown with SD and low glucose medium

Caloric restriction (CR) is well known to be a reliable means of extending the lifespan of yeast, worms, flies, rodents, and potentially even nonhuman primates. Many NAD^+^‐dependent HDACs are involved in mediating lifespan extension, repressing the expression of particular genes in response to CR (Imai et al., 2000). Consistent with this model, we hypothesized that HATs may play an important role in this process based upon their regulation of energy homeostasis (Houtkooper, Pirinen, & Auwerx, 2012). To test this, we treated *ngg1Δ*, *gcn5Δ,* and BY4741 cells in either normal SD media (2% glucose) or 0.5% glucose SD media, revealing that this low glucose treatment significantly increased BY4741 lifespan and reduced cell cycle length, whereas no such effect was observed in *gcn5Δ* or *ngg1Δ* cells (Figure 4a, Figure S4a–c), thus indicating that this CR‐associated lifespan extension is GCN5‐ and NGG1‐dependent. Western blotting further confirmed that CR was associated with significantly reduced H3K9 and H3K18 levels, as was observed in *gcn5Δ, ngg1Δ, GCN5gcn5*, and *NGG1ngg1* strains (Figure 4b,d). We also observed significant lifespan extension in BY4743, *GCN5gcn5,* and *NGG1ngg1* strains following treatment with low glucose media, as expected (Figure 4c). We further found that CR‐associated lifespan extension was abolished in H3K9A and H3K18A cells, but not in H3K14A mutants (Figure 4e–g), consistent with CR acting through GCN5 and disrupting H3K9/K18 acetylation in order to extend longevity. Inhibiting GCN5 therefore mimics CR and thereby extends lifespan.

### 2.5 Identification of pathways and genes induced downstream of HAT inhibition

In an effort to identify the genes and pathways induced upon GCN5 inhibition, we first assessed gene expression profiles in our treated samples (Table S1) and compared them to those of profiles from samples under specific conditions (http://genome.ucsf.edu/CSEPCT/). This analysis revealed that our treated samples resembled samples that had undergone heat shock (http://www.ncbi.nlm.nih.gov/geo/query/acc.cgi?acc=GSE38478, time point 5, *r* = .234, *p‐*value < 3.02e−57), with motif enrichment analyses suggesting the activation of the Msn2/4 transcription factor, as shown in Table S2. Msn2/4 is a transcription factor essential for lifespan extension associated with caloric restriction in yeast, and heat shock factor Hsf‐1 overexpression can prolong the lifespan of worms (Hsu, Murphy, & Kenyon, 2003). The gene expression profile was also negatively correlated with that of an aging time course in *Caenorhabditis elegans* (day 13 http://www.ncbi.nlm.nih.gov/geo/query/acc.cgi?acc=GSE12094, http://www.ncbi.nlm.nih.gov/geo/query/acc.cgi?acc=GSE12168, *r* = −.196, 899 ortholog pairs, *p‐*value < 3.26e−9) based on a comparison of homologous genes, consistent with the hypothesis that EGCG induces a gene expression profile that reverses the normal aging processes. This profile was also associated with shifts in the Dauers versus Mix‐Stage (http://www.ncbi.nlm.nih.gov/geo/query/acc.cgi?acc=GSE30977, http://www.ncbi.nlm.nih.gov/geo/query/acc.cgi?acc=GSE36644), consistent with aging arrest in worms and further supporting the notion that EGCG facilitates the rejuvenation of treated cells.

**Figure 5:**
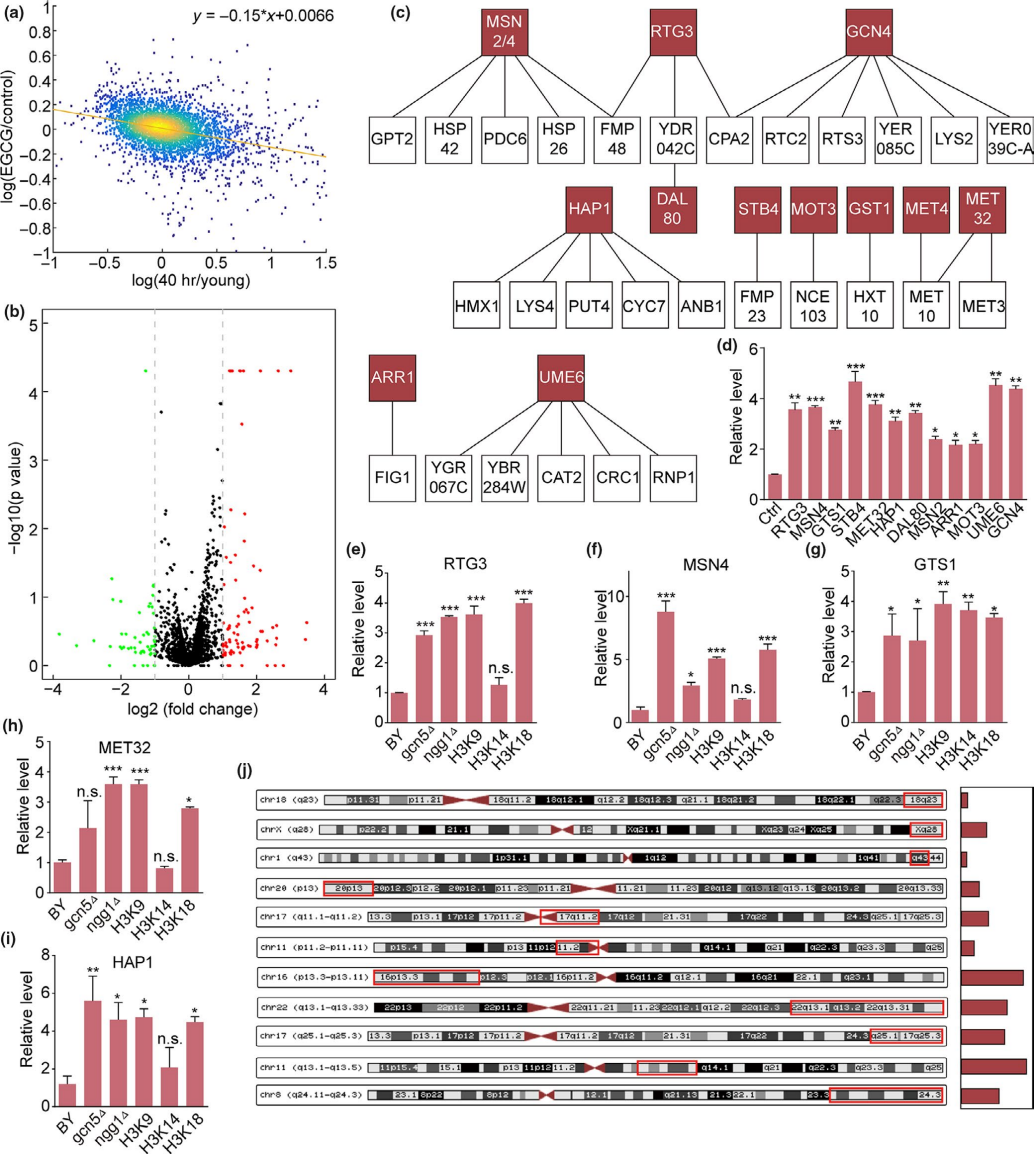
The gene expression landscape induced by GCN5 inhibition. (a) Correlation analysis of transcriptomes in EGCG‐treated cells and 40‐hr aged yeast population (*r* = −.36, *p*‐value < 8e−137). (b) A volcano plot of EGCG‐mediated changes in gene expression. (c) Genes up‐ or down‐regulated by EGCG are shown. (d) RTG3, MSN4, GST1, STB4, MET32, HAP1, MSN2, ARR1, MOT3, UME6, and GCN4 mRNA levels in yeast following a 24‐hr EGCG treatment. (e–i) mRNA levels of RTG3 (e), MSN4 (f), GST1 (g), MET32 (h), and HAP1 (i) in WT, *gcn5Δ*, *ngg1Δ*, H3K9A, H3K14A, and H3K18A cell strains. (j) Map of the positions of upregulated genes in human cell lines treated with EGCG (red boxes). Regions were located in subtelomeric or subcentromeric regions, with the number of genes indicated by bars next to each chromosome

In another independent analysis, we compared the transcriptional changes following EGCG and GA treatment with those observed in yeast aging (Hendrickson et al., 2018). We found that EGCG reversed the aging transcriptome at the genome‐scale level (*r* = −.36, *p*‐value < 8e−137 for EGCG vs. 40 hr aged yeast population, Figure 5a). The same result was also observed for GA (Figure S5a). In *gcn5∆*, such a correlation was absent (Figure S5b). Hendrickson's results also revealed that stress genes were involved in yeast aging, consistent with our analysis aforementioned.

Genes that were upregulated upon EGCG treatment were enriched for those genes linked to lysine synthetic pathways, thiamine metabolism, and sulfur metabolism based on a transcription factor enrichment analysis (Figure 5b). In this analysis, we found RTG3, MSN4, GTS1, MET32, STB4, HAP1, MET4, DAL80, MSN2, MOT3, ARR1, UME6, and GCN4 to be enriched in our treated samples (Figure 5c,d). We also observed the upregulation of two genes downstream of IME1, which, together with UME6, is a meiotic regulator. Overexpressing these meiotic transcription factors has been linked to sporulation‐associated lifespan extension in old cells (Unal, Kinde, & Amon, 2011). RTG3, MSN4, GTS1, MET32, and HAP1 were overexpressed in *ngg1Δ*, *gcn5Δ*, H3K9A, and H3K14A strains (Figure 5e–i, Figure S5c–j) as measured via RT‐PCR. Rtg3 is a bHLH/Zip transcription factor involved in the retrograde (RTG) and TOR pathways, which, as part of a complex with the bHLH/Zip protein Rtg1p, activates those two pathways. RTG3 is also known to regulate the replicative lifespan of cells (Jiang et al., 2016; Miceli, Jiang, Tiwari, Rodriguez‐Quinones, & Jazwinski, 2011). These results thus indicate that inhibitor treatment induces multiple protective pathways in treated cells.

To determine how EGCG influences downstream genes in human cells, we analyzed a dataset from human bronchial epithelial cells treated for 6 hr with EGCG using a Gene Set Enrichment Analysis (GSEA) approach (Subramanian et al., 2005). This revealed that many of the upregulated genes were enriched at subtelomeric regions or were near to centromeres, as in 18q23, Xq28, 1q43, 20p13, 17q11.2, 11p11.2, 16p13.3, 22q13.1, 22q13.31, 25q25.1, 17q25.3, and 8q24.3 (Figure 5j, Figure S6).

To determine which transcription factors regulate these genes, we compared our dataset to motif sets, revealing these genes to be regulated by SP1 (271 genes), LFA1 (213 genes), STAT1 (56 genes), TEL2 (193), ELK1 (204), USF1 (239), CREB (230,) MIR503 (22), and other unidentified binding motifs such as CTGRYYYNATT (86). Of the identified genes, LFA1 and TEL2 are located at end of chromosomes 21 and 16, respectively, confirming the fact that subtelomeric regions are being activated. The SP1 transcription factor is linked to breast cancer and Huntington's disease, acting together with ATF7IP to maintain telomerase activity via inducing the expression of TERT and TERC. Together, our results thus suggest that HAT inhibition alters the expression of subtelomeric genes, leading to the rejuvenation and lifespan extension of senescent cells.

## 3 DISCUSSION

The yeast cells on our U‐shape chip suffered from greater pressure and thus had lives that were 2–3 generations shorter than those on island chips. However, as shown in this study, our published work (Dang et al., 2019) and in other unpublished data, we observed conserved effects of EGCG, AA, berberine, and approximately 10 total compounds in both yeast and human cell lines. As the lifespan of yeast is limited to around 25 generations by ERCs, we postulate that the use of a U‐shape chip and SD media offers greater opportunity for lifespan extension than do traditional approaches. Because the lifespan of wild‐type yeast is negatively correlated with flow pressure and may thus vary between experiments, we utilized a paired dataset in order to ensure that each pair of treated and control samples was exposed to comparable conditions. We therefore believe that the use of yeast in such a U‐shape chip represents a suitable model system for anti‐aging drug screening.

Many studies have shown that the overexpression of the NAD^+^‐dependent sirtuins is sufficient to extend the lifespans of budding yeast, *C. elegans*, and *D. melanogaster* (Viswanathan, Kim, Berdichevsky, & Guarente, 2005). In contrast, at the cellular level suppressing sirtuins expression is associated with accelerated aging in an H4K16ac‐dependent fashion (Dang et al., 2009). The development of drugs targeting sirtuins is thus not ideal. To better explore the full landscape of these relationship between acetylation and aging, we therefore elected to study HATs, which function in a manner opposite to that of sirtuins.

*Saccharomyces cerevisiae* serve as an ideal model for the study of aging owing to their short life cycle. Using a single‐cell microfluidic approach, we evaluated the effect of HAT inhibitors on yeast lifespan. Telomeric chromatin region regulation is known to be linked to the posttranslational modification of histones (Blasco, 2007). Inactivation of the HAT Sas2 has been shown to delay the onset of senescence in yeast lacking telomeric function (*tlc1Δ*) via a mechanism dependent upon homologous recombination (Kozak et al., 2010). We explored the role of nonessential HATs in lifespan extension, revealing Gcn5 to be a key regulator of these processes. Gcn5 is a SAGA catalytic subunit necessary for activating transcription and lifespan extension in the retrograde response (Jiang et al., 2016). Yeast which lack GCN5, however, do not exhibit an extended lifespan, and as such, we generated heterozygous yeast with one copy of GCN5, leading to an extension of the replicative lifespan. When forming the catalytic subunits of ADA and SAGA, Gcn5 associates with Ada2 and Ngg1. As it possesses an acetyl‐lysine‐binding bromodomain, Gcn5 regulates the acetylation of lysosomes and certain promoter regions in cells. Gcn5 cannot mediate nucleosome acetylation without the rest of the SAGA complex, and consistent with this, we found that NGG1 knockdown cells exhibited similar or even improved lifespan extension compared to GCN5 knockdown cells.

The ADA complex acetylates H3 lysine residues in the following order of preference: H3K14 > H3K23 > H3K9 ≈ H3K18 > H3K27 > H3K36. When Gcn5 is defective in this complex, studies have shown the presence of site‐specific changes in consequent acetylation, with particularly marked reductions in H3K18Ac (Cieniewicz et al., 2014). We found that *gcn5Δ* and *ngg1Δ* cells exhibited a loss of H3K9 and H3K18 acetylation without any significant changes in H3K23 or H3K36 levels. We also determined that mutating these K9 and K18 residues disrupted the EGCG‐mediated lifespan extension effect, suggesting that these H3 residues are the primary targets of GCN5.

Generally, senescence is believed to be caused by telomere shortening. Since we found that HAT inhibition was sufficient to rejuvenate cells, it is possible that this effect was related to telomeres/telomerase activity. Because we also found that HAT inhibition alleviated SA‐β‐gal staining, which is a hallmark of aging, this further suggests that HAT inhibition likely functions downstream of the telomere pathways.

While much research has focused on caloric restriction (CR), its mechanisms are poorly understood. SIRT1 and its homologs have been shown to be a key downstream protein mediating the effects of CR in yeasts, rodents, and humans (Cohen et al., 2004). Indeed, compounds that activate SIRT1 can recapitulate many molecular signaling events associated with CR in vivo, enhancing metabolic signaling, decreasing inflammation, and increasing mitochondrial biogenesis (Smith et al., 2009). Many studies have clearly shown that CR impacts chromatin structure and function (Vaquero & Reinberg, 2009), but studies have not firmly established whether specific CR‐associated changes in histone modification are linked to increased longevity.

Because the acetylation substrate acetyl‐CoA is synthesized from glucose through glycolysis, low glucose likely causes lower levels of acetylation. We thus hypothesized that CR may act through histone acetylation. Indeed, we found that CR significantly impaired overall H3, H3K9, and H3K18 acetylation, much as was observed upon Gcn5 inhibition. CR‐associated extension of cellular lifespan was abolished in *gcn5Δ* yeast, consistent with the results in lines with mutant isoforms of H3K9 and K18 residues. We were able to demonstrate a novel mechanism whereby CR targets Gcn5‐mediated histone H3 acetylation, thereafter regulating stress response pathways and subtelomeric genes to extend lifespan. Given such evidence of similarity between the inhibition of HAT and CR, this may represent an effective means of mimicking CR. The administration of HAT inhibitors may allow one to achieve the benefits of CR without the need for starvation.

KAT6A/B inhibitors have been reported to promote senescence (Baell et al., 2018), unlike our GCN5 findings. This may be because KAT6A/B are MYST‐related HATs, whereas we were focused on GCN5‐related N‐acetyltransferases (GNATs). MYST acetyltransferases have a specific conserved HAT domain (Avvakumov & Cote, 2007). KAT6A/B‐mediated senescence is dependent upon INK4A/ARF, whereas GCN5 does not regulate these proteins. Given that KAT2A (GCN5) is distantly related to KAT6A/B and that they regulate different pathways, our results are compatible with previous findings for KAT6A/B.

Epigallocatechin gallate increases the lifespan of rats, and this effect is attributed to its antioxidative and anti‐inflammatory properties (Niu et al., 2013). In this novel study, we chose to focus more specifically on the efficacy of EGCG as a HAT inhibitor, and we demonstrated that the prolongation of lifespan by acetyltransferase inhibition is more significant than that achieved via the use of antioxidants. Our results using human cell lines further indicated that HAT inhibitors are effective in different species, highlighting the great potential of HAT inhibitors as anti‐aging drugs.

There are certain limitations to our present study. For one, GCN5‐knockout mice die during the embryonic period, limiting our ability to assess the role of this protein in vivo. In addition, more data are needed regarding how CR influences aging and to what extent this effect is dependent upon Gcn5 and Ngg1. Both energy metabolism and TOR signaling may also be involved in this signaling and associated phenotypes.

**Figure 6:**
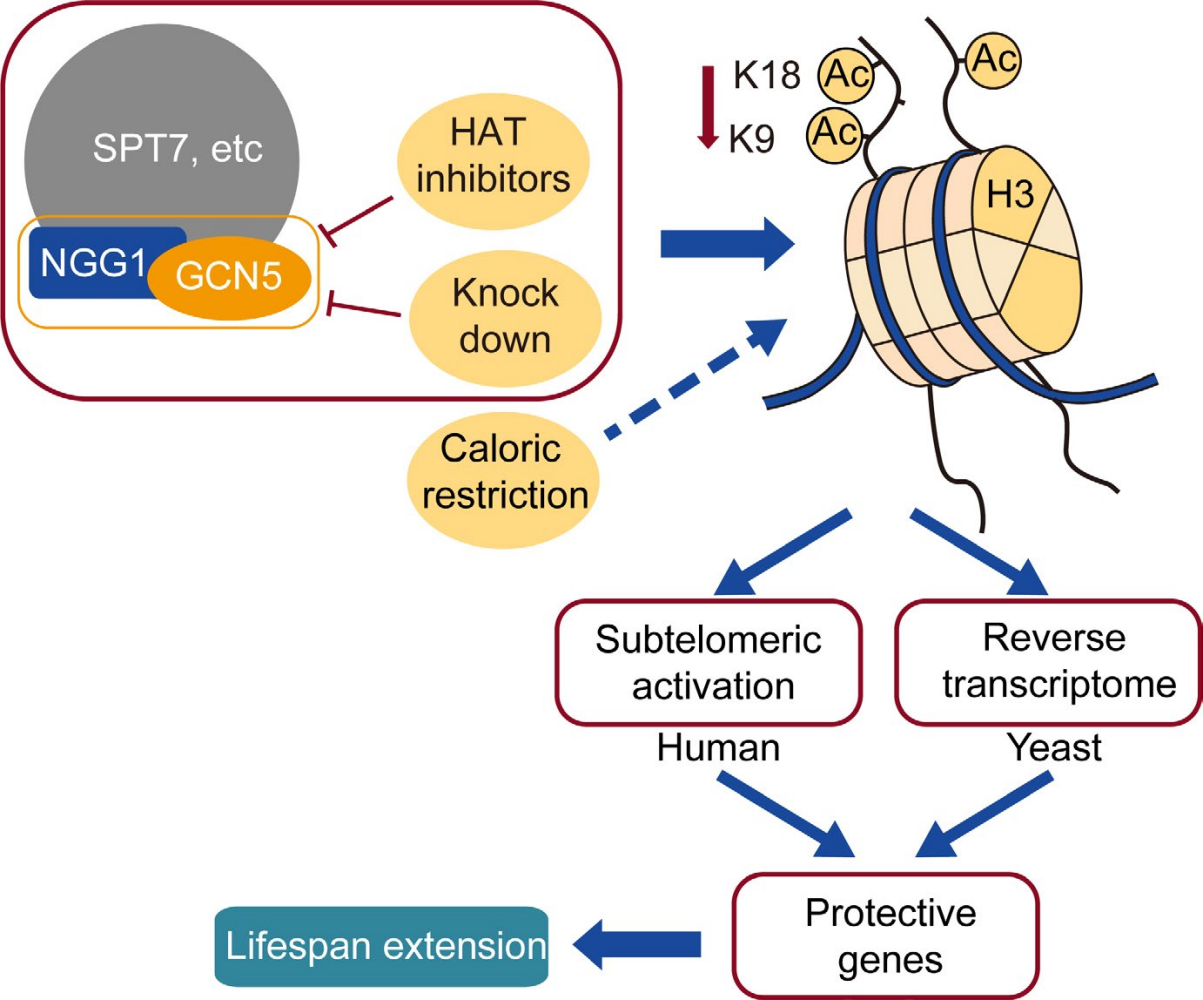
A model depicting how the inhibition of histone acetyltransferase GCN5 extends lifespan

In summary, we generated a GCN5 knockdown model system and confirmed the importance of Gcn5 inhibition as a means of extending cellular lifespan. We further revealed that CR is able to increase longevity in a manner dependent upon Gcn5, in concert with Ngg1, leading to modulations in H3K9/K18 acetylation, thereby affecting the expression of specific genes associated with aging (Figure 6). Efforts to target this pathway via regulating GCN5 activation may therefore represent a means of simulating CR and thereby combatting the aging process.

## 4 MATERIAL AND METHODS

### 4.1 Yeast strain generation

Standard SD media (1% yeast extract, 1% bacto‐peptone, 2% glucose) was used for routine growth of all yeast strains at 30°C on a 250‐rpm rotary shaker. Experiments designed to assess yeast lifespan were performed using yeast in the logarithmic phase of growth via growing yeast overnight and then diluting them 4 hr before use.

Yeast strains bearing the substitution mutations H3K9A, H3K14A, H3K14Q, H3K23A, H3K23R, and H3K23Q, as well as the wild‐type H3 control strain, were obtained from the H3‐H4 histone mutant library (Dai, et al., Cell, 2008). Yeast strains bearing the substitution mutations H3K18A, H3K18R, and H3K18Q, as well as a wild‐type control strain, were generated via the standard gene replacement procedure by replacing the (hht1‐hhf1)∆::LEU2 (hht2‐hhf2)∆::HIS3 alleles in FY1716 (a gift from Fred Winston) with corresponding mutant or wild‐type alleles of HHT1‐HHF1 and HHT2‐HHF2. The wild‐type allele of HHT1‐HHF1 was cloned and maintained in the plasmid pWD120, derived from pRS314. The wild‐type allele of HHT2‐HHF2 was obtained from the plasmid pRM204. H3 substitution mutations in these plasmids were generated by site‐directed mutagenesis and verified by sequencing. All DNA fragments used for gene replacements were produced by PCR from corresponding mutant‐containing plasmids. All yeast strains used are listed in Table S5.

### 4.2 Microfluidics analysis

We used repeated microscopy in order to monitor mother cells over a 2‐day period as in previous reports (Xie et al., 2012; Zhang et al., 2012) and a modified U‐shape derived from (Casavant et al., 2013). This approach relied upon the use of microposts or a jail in the microfluidic system that maintained mother cell position while allowing for the hydrodynamic flow‐mediated removal of daughter cells. Epigallocatechin gallate (Sigma), AA (Sigma), garcinol (GA) and curcumin were dissolved in DMSO and then diluted in media to 10 μg/ml.

### 4.3 Western blot

WI‐38 and 2BS cells were homogenized in RIPA lysis buffer (Thermal Scientific) supplemented using a protease inhibitor cocktail (Roche). A Dounce homogenizer was used to homogenize these protein extracts prior to spinning for 20 min at 12,000 *g* at 4°C. Total protein extraction kits for microbes with thick cell walls (Invent) were used when extracting protein from yeast. A BCA assay (Pierce) was used for protein quantification, after which proteins were separated with SDS‐PAGE gels and transferred onto PVDF membranes (Amersham Biosciences). Blots were then probed using polyclonal antibodies against H3 (Active motif), H3‐Ac (Active motif), H4 (Active motif), H4‐Ac (Active motif), H3K9Ac (Active motif), H3K14Ac (Active motif), H3K18Ac (Active motif), H3K23Ac (Active motif), H3K36Ac (Active motif), Ac‐lysine (Active motif), and β‐tublin (Proteintech). Secondary goat anti‐rabbit or anti‐mouse IgG (Scicrest) was then used to probe blots, which then underwent development using an ECL Plus Kit (Amersham Biosciences). The optical density of each band was used as a means of quantifying relative protein levels.

### 4.4 Cell culture

WI‐38 and 2BS cells were provided by the National Institutes for Food and Drug Control. Cells were counted and seeded at equal densities for every passage and were grown in MEM (Gibco) supplemented with 10% FBS (Gibco) in an incubator at 37°C and 5% CO_2_. Epigallocatechin gallate (Sigma) and AA (Sigma) were dissolved in DMSO and then diluted in media to 10 μg/ml. This drug‐treated media was then used to treat cells for a 24‐hr period, replacing the unsupplemented culture media.

### 4.5 Cell cytotoxicity assays

Cytotoxicity was assessed via the CCK‐8 assay kit (Dojindo). Briefly, we plated 2BS or WI38 cells into 96‐well plates (5000/well) and then treated them for 24 hr with EGCG. Next, we added 1:10 dilution of CCK‐8 solution in culture media for 1 hr at 37°C. A microplate reader (Biotek, MQX200) was then used to measure absorbance at 450 nm.

$$\text{Cell viability (}\%)=\left({\text{OD}}_{\text{treatment}}-{\;\text{OD}}_{\text{blank}}\right)/\left({\text{OD}}_{\text{control}}-{\;\text{OD}}_{\text{blank}}\right)\;\times \;100.$$

### 4.6 Cell growth assay

The CCK‐8 kit was also used as a means of assessing the proliferation of cells*,* which were plated (2,000/well) in a 96‐well plate. Cells were then treated for 1 week using EGCG and AA (2.5 mol/ml for EGCG; Sigma and 10 mol/ml for AA; Sigma). Media was refreshed daily with fresh ECGC, AA, or DMSO. At appropriate timepoints, we harvested five replicate wells per condition and treated them with CCK‐8 solution as above for 1 hr and measured absorbance at 450 nm. Curve generation was repeated twice. For 1‐month long assessments of proliferation, cells were instead plated in 6‐well plates at 2 × 10^6^ cells per well and treated using EGCG, with cells being harvested and counted after 4 days.

### 4.7 RNA extraction and quantification

TRIzol was used to lyse cells, followed by RNA extraction using isopropanol and chloroform. We then used a total of 1 μg RNA for cDNA reverse transcription using a cDNA synthesis kit (Abmgood). We then used real‐time qPCR to assess the expression of genes of interest, normalizing expression levels to GAPDH. All primers are listed in Table S4.

### 4.8 Senescence‐associated β‐galactosidase (SA‐β‐gal) staining

We washed cells in PBS and then fixed them for 5 min with 4% formaldehyde at room temperature, followed by an additional wash. Cells were then stained at 37°C overnight using staining buffer (1 mg/ml 5‐bromo‐4‐chloro‐3‐indolyl‐β‐d‐galactopyranoside (X‐gal), 40 mM citric acid/sodium phosphate, pH 6.0, 5 mM potassium ferrocyanide, 5 mM potassium ferricyanide, 150 mM NaCl, 2 mM MgCl_2_) and were then analyzed based on provided instructions (CST, 9860S).

### 4.9 GCN5 and NGG1 gene silencing

To silence GCN5 and NGG1, INTERFERin (Polyplus Transfection) was used to transfect cells with appropriate siRNAs. Human siRNAs against GCN5 and NGG1 were prepared by GenePharma (Shanghai), with the following sequences: *gcn5* siRNA1 5′‐CCCUGGAGAAGUUCUUCUATT‐3′; *gcn5* siRNA2 5′‐CAGCCCUCCAUUUGAGAAATT‐3′; NGG1 siRNA1 5′‐GGAAGGGAAGGCAGGACAUTT‐3′; NGG1 siRNA2 5′‐GGACACUAAAGAUGUGGAUTT‐3′.

### 4.10 EGCG and Gcn5 interaction assay

The DNA sequence encoding the HAT domain of yGCN5 (99‐262) was amplified by PCR and was sub‐cloned into a pET‐28a vector for overexpression. The plasmid was transformed into the *Escherichia coli* strain BL21(DE3) and was overexpressed by induction with 0.5 mM isopropyl *β*‐d‐thiogalactoside and incubated at 15℃. The protein was purified via sequential use of Ni‐NTA resin (Merck Millipore) and Superdex 75 (GE) gel filtration chromatography. Purified protein was concentrated to 10 mg/ml by using a Centriprep 10 concentrator. 50 μM Gcn5 protein (residues 99‐262) in 20 mM sodium phosphate, 0.15M NaCl, and pH8.0 was incubated with EGCG at room temperature for 30 min at different concentrations, and then, the mixtures were analyzed by SDS‐polyacrylamide gel electrophoresis (SDS‐PAGE).

### 4.11 Statistical analyses

The Wilcoxon rank‐sum test was used to compare lifespan differences, whereas changes in cell cycle length were assessed via *F* tests. Western blotting results were assessed by two‐tailed Student's *t* tests or ANOVAs to compare two and more than two samples, respectively. Data are means ± s.e.m. *p* < .05 was the significance threshold. *p < .05, **p < .01, ***p < .001. n.s., not significant. Results were based on a minimum of three independent experiments unless otherwise indicated. GraphPad Prism was used for statistics unless otherwise indicated. Cell numbers, mean lifespan, comparative *p‐*values, and type of chip used were listed in Table S3.

### 4.12 RNA‐seq

Yeast were grown in SD medium and treated with EGCG or GA for 12 hr before harvest. Total RNA isolation was then performed using the MasterPure Yeast RNA Isolation Kit from Epicentre Biotechnologies (# MPY03100) following the manufacturer's instructions. All RNA fragments were converted into deep‐sequencing libraries using TruSeq Stranded Total RNA Library Prep Kit with Ribo‐Zero Gold (#RSS‐122‐2301, Illumina). The Ambion ERCC RNA spike‐in control (#4456740, Life Technology) was included in the library construction.

## CONFLICT OF INTEREST

The authors declare that they have no conflict of interest.

## AUTHORS' CONTRIBUTION

D.Z., B.H., Z.X., and B.Y. conceptualized the project. D.Z. and B.H. were involved in designing and conducting the experiments. Y.A., J.Z., and M.G. assisted with microfluidics analyses. S.Z. and M.W. assisted with cell culture. D.Z., B.H., Z.X., and W.D. analyzed data. D.Z., B.H., and Z.X. prepared the draft with input from all authors. Z.X. and B.Y supervised research. All co‐authors contributed substantially to this manuscript.

## Supporting information


**Figures S1‐S6**

**Table S1**

**Table S2**

**Table S3**

**Table S4**

**Table S5**
