# Supplementary material for: Inhibition of histone acetyltransferase GCN5 extends lifespan in both yeast and human cell lines
Source: Aging Cell. 2020 Mar 11;19(4):e13129. doi: 10.1111/acel.13129 (PMC7189995; doi:10.1111/acel.13129)
Supplement: Supplementary file 1 — Figures S1‐S6 [file ACEL-19-e13129-s001.docx]

**Supplementary Information**


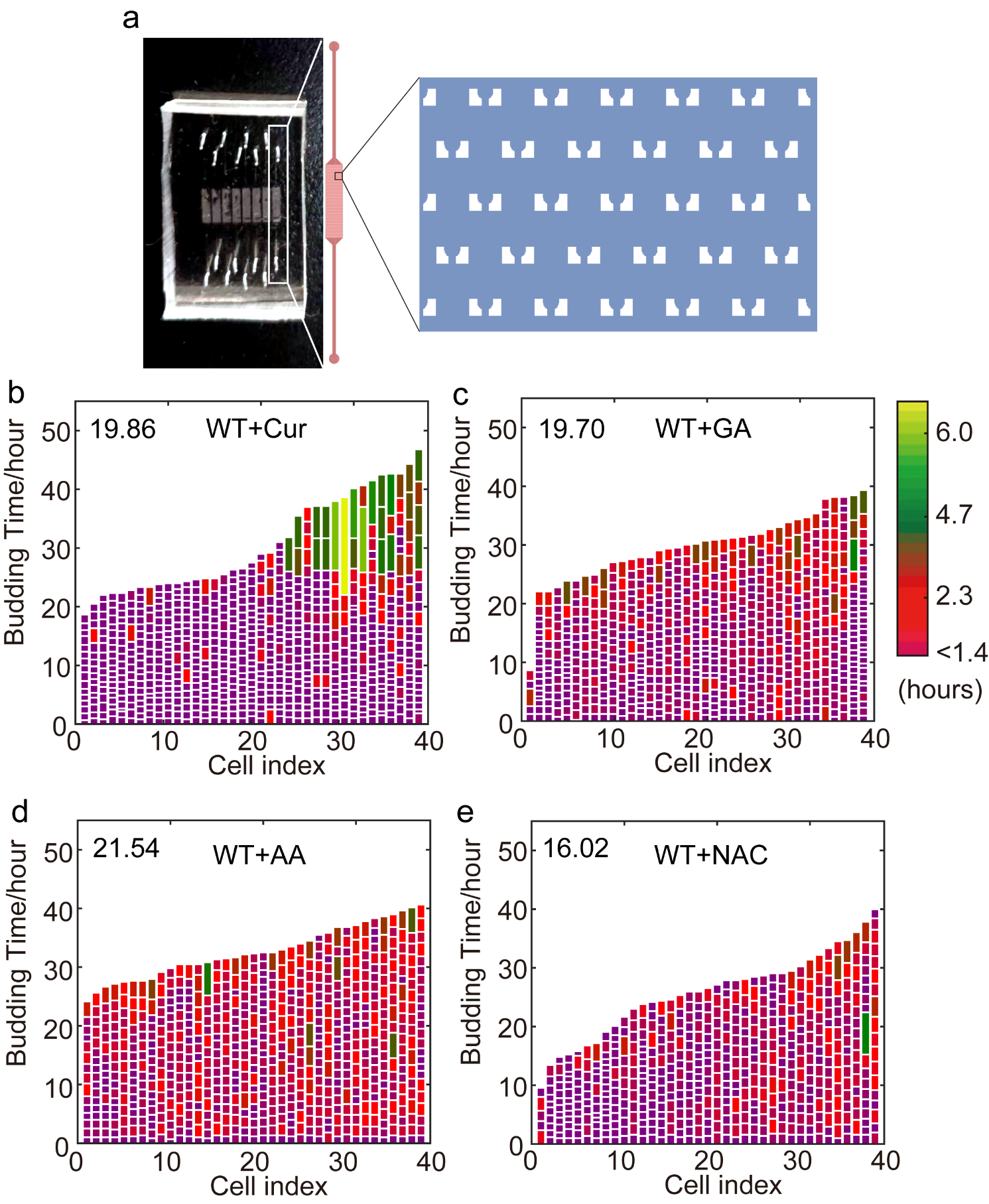


**Supplementary Figure 1| Effect of different HAT inhibitors on yeast replicative lifespan. a,** the inside construction schematic drawing of the modified from U-shape chip. **b-e,** mother cell budding profiles for (**b**) WT+Cur, (**c**) WT+GA, (**d**) WT+AA, (**e**) WT+NAC (Exponential color scale; cell cycles with durations 1.4 h or less were colored in purple, number of cells [n]: 37).


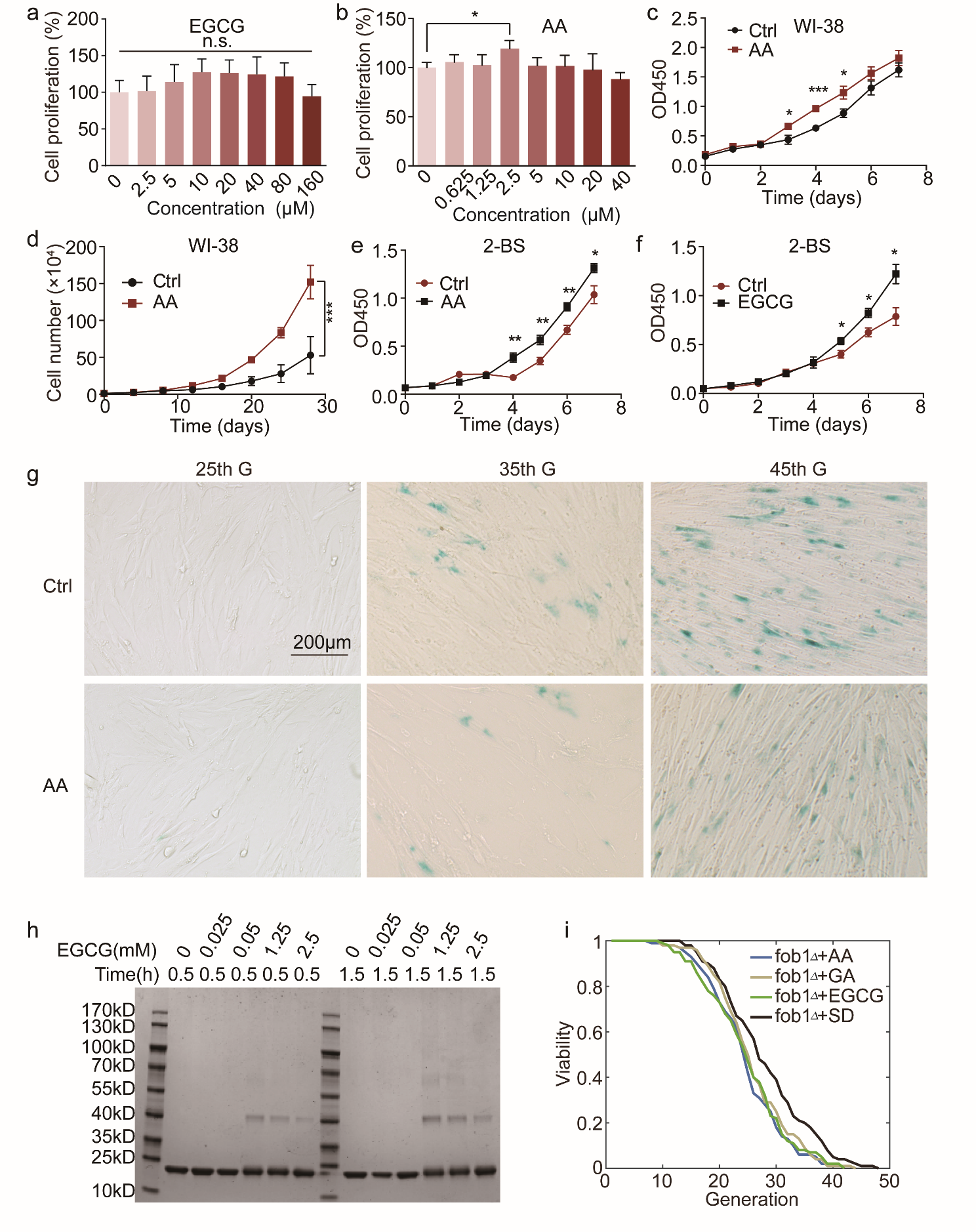


**Supplementary figure** **2| HAT inhibitors extends lifespan of human cell lines. a,** CCK8 level of different concentration of EGCG administration in WI-38 cells. **b,** CCK8 level of different concentration of AA administration in WI-38 cells. **c,** effects of AA on cell division curve for over a week in WI-38 cells. **d,** effects of AA compared with the DMSO vehicle control on cell growth of WI-38 cells lasting for one month. **e,** effect of AA on cell division curve for over a week in 2-BS cells. **f,** effect of EGCG on cell division curve for over a week in 2-BS cells. **g,** SA**-**β-gal staining of WI-38 cells for 3 periods after treatment with 2.5 µM AA or DMSO vehicle control. **h,** the EGCG and Gcn5 covalently bond split band in SDS-PAGE. **i,** HAT inhibitors did not extend the lifespan of *fob1∆* further as compared to cells on SD media. Student’s t-test (**c, d, e, f**), one-way ANOVA test (**a, b**).


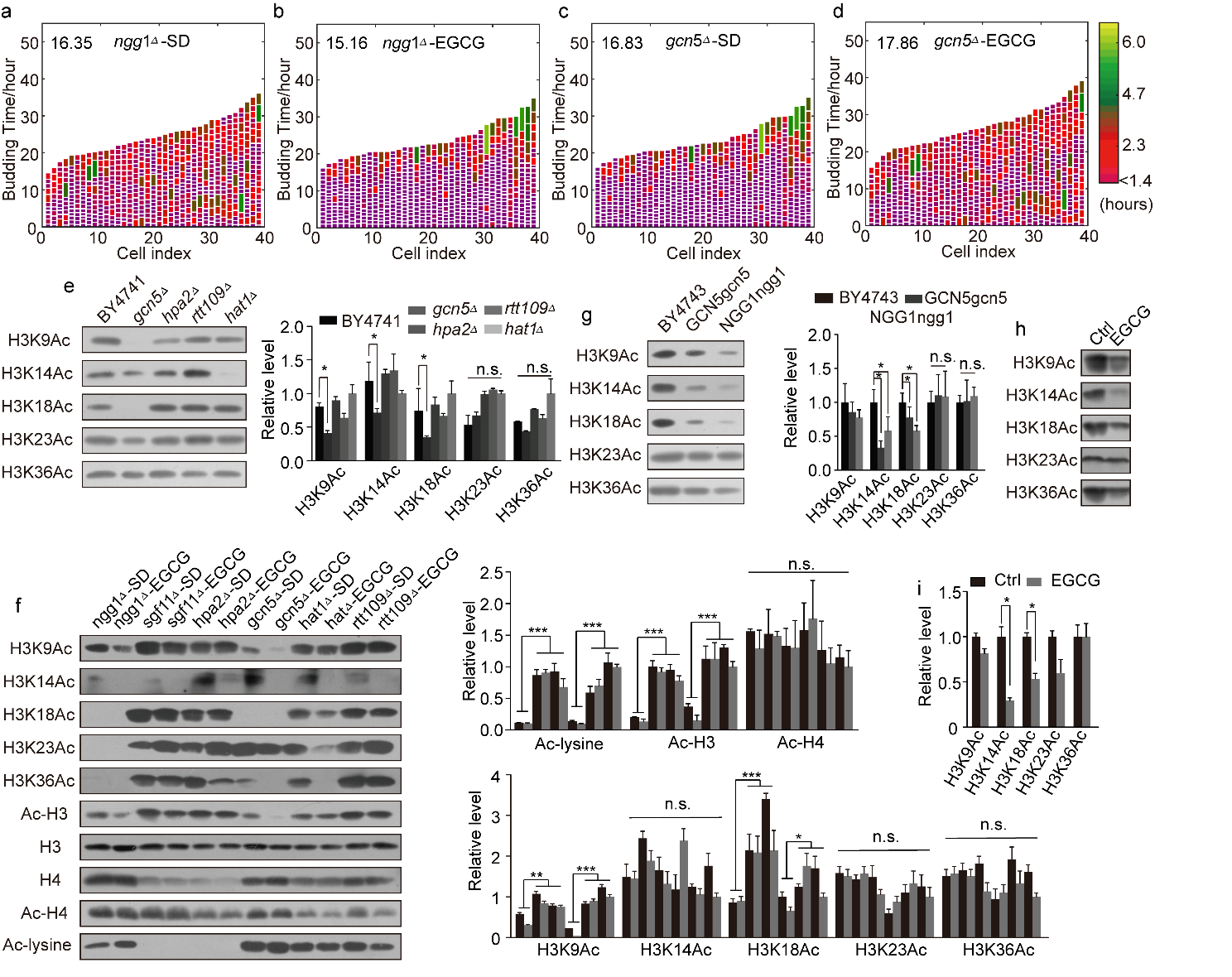


**Supplementary figure 3| Replicative lifespan and histone acetylation level of mutation cell strains. a-d,** mother cell budding profiles for (**a**) *ngg1Δ*+SD, (**b**)*ngg1Δ*+EGCG, (**c**) *gcn5Δ*+SD, (**d**) *gcn5Δ*+EGCG. (Exponential color scale; cell cycles with durations 1.4 hr or less were colored in purple, number of cells [n]: 37). **e,** expression of H3K9Ac, H3K14Ac, H3K18Ac, H3K23Ac and H3K36Ac of BY4741, *gcn5Δ*, *hpa2Δ*, *rtt109Δ*, and *hat1Δ* cell strains by Western blot; statistics of Western blot (n=3). **f,** expression of H3K9Ac, H3K14Ac, H3K18Ac, H3K23Ac, H3K36Ac, Ac-H3, H3, H4, Ac-H4 and Ac-lysine of different subunits of SAGA and ADA knockout cell strains in SD or EGCG medium by Western blot; statistics of Western blot (n=3). **g,** expression of H3K9Ac, H3K14Ac, H3K18Ac, H3K23Ac and H3K36Ac in BY4743, GCN5gcn5 and NGG1ngg1 cells; statistics of Western blot (n=3). **h,** expression of H3K9Ac, H3K14Ac, H3K18Ac, H3K23Ac and H3K36Ac of cells in SD and EGCG medium by Western blot. **i,** statistics of Western blot (n=3). One-way ANOVA test (**e**-**h**)


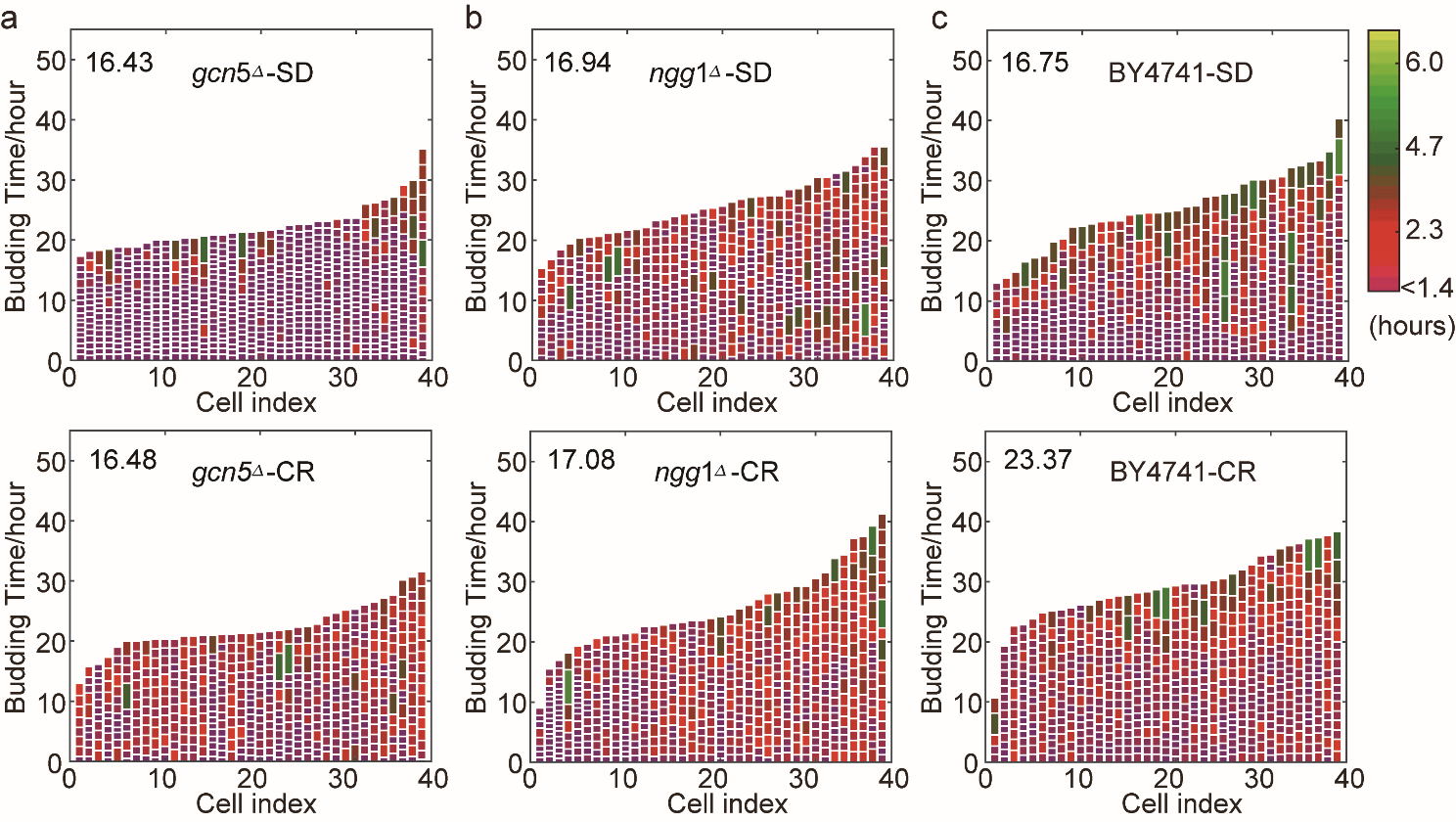


**Supplementary figure 4| Lifespan of WT, *ngg1Δ*, *gcn5Δ* on SD or CR medium. a-c,** mother cell budding profiles for (**a**) *gcn5Δ* in SD or CR medium, (**b**) *ngg1Δ* in SD or CR medium, (**c**) BY4741 on SD or CR medium. (Exponential color scale; cell cycles with durations 1.4 hr or less were colored in purple, number of cells [n]: 37).


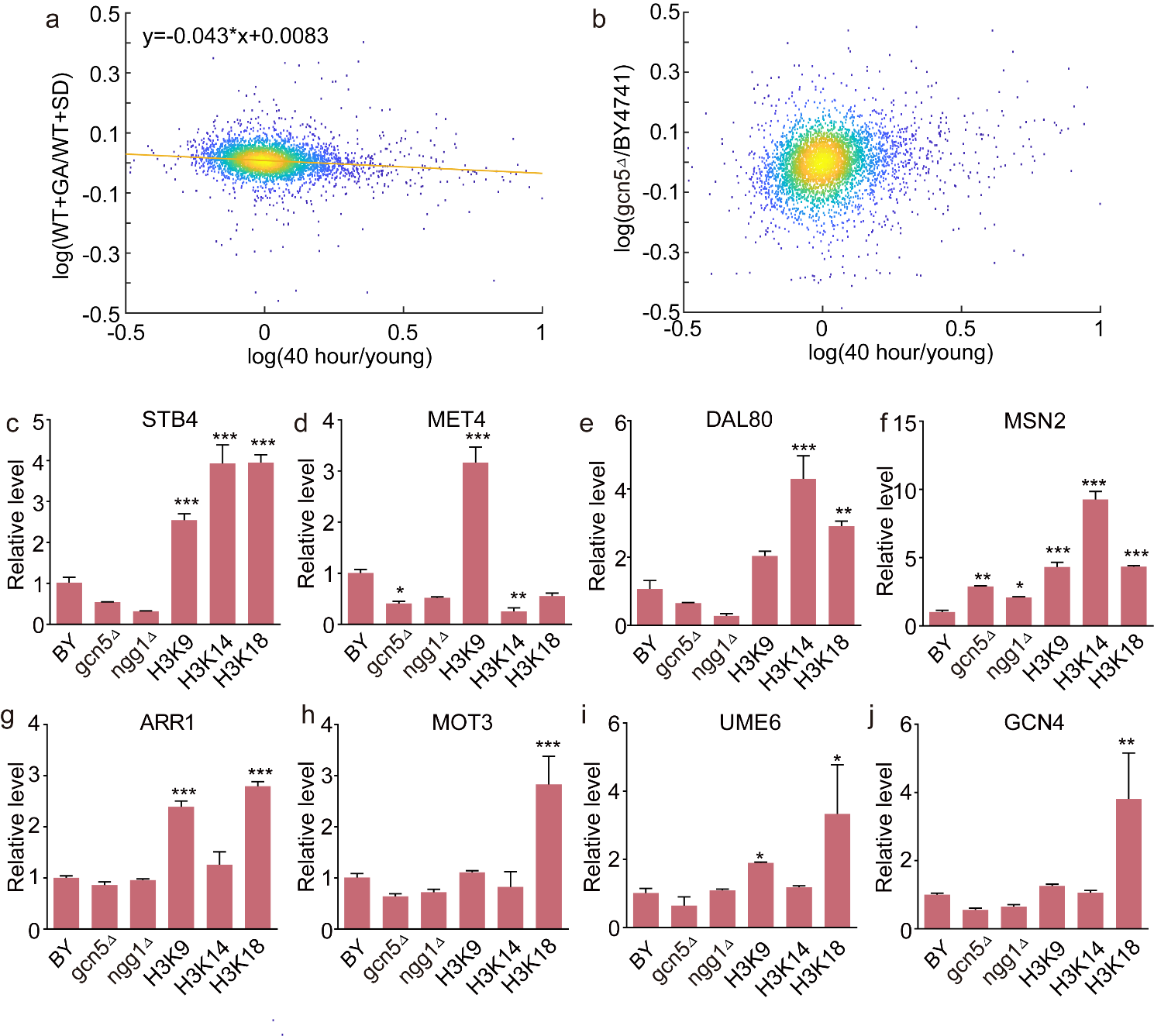


**Supplementary figure 5| Gene transcription level and effect of CR on replicative lifespan of mutation cell strains. a,** Correlation analysis of transcriptomes in GA treated cells versus 40 hours aged yeast population. **b,** Correlation analysis of transcriptomes in GA treated cells versus 40 hours aged yeast population in *gcn5Δ*. **c-j,** mRNA levels of STB4 (**c**), MET4 (**d**), DAL80 (**e**), MSN2 (**f**), ARR1 (**g**), MOT3 (**h**), UME6 (**i**), GCN4 (**j**) in WT, *gcn5Δ*, *ngg1Δ*, H3K9A, H3K14A, H3K18A cell strains. One-way ANOVA test (**c**-**j**).


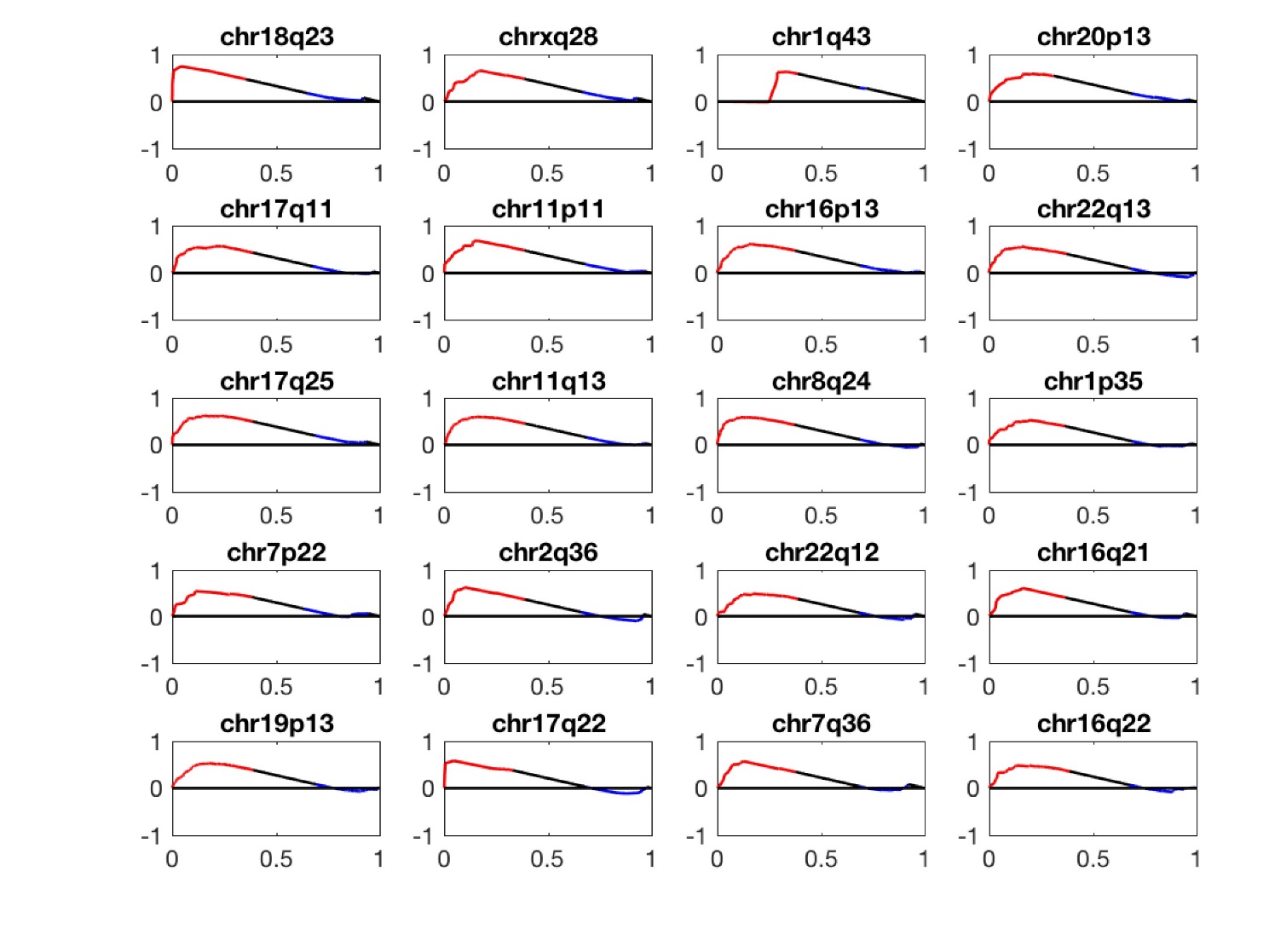


**Supplementary figure 6|** **Gene Set Enrichment Analysis (GSEA) of upregulated genes.** Curves are the running sum for S (>0 in red, =0 in black and <0 in blue) in the data set. The maximum running sum is the enrichment score (ES).

**Supplementary Table 1|** **RNA-seq of BY4741+SD, BY4741+EGCG, BY4741+GA, *gcn5Δ*+SD, *gcn5Δ+EGCG*.**

**Supplementary Table 2|** **Hypergeometric analysis for transcription factors.**

**Supplementary Table 3| Cell numbers, mean lifespan, comparative p-values and type of chip associated with lifespan curves.**

**Supplementary Table 4| Primers used for RT-PCR.**

**Supplementary Table 5| The information of yeast strains used.**
