# Supplementary material for: Inhibition of histone acetyltransferase GCN5 extends lifespan in both yeast and human cell lines
Source: Aging Cell. 2020 Mar 11;19(4):e13129. doi: 10.1111/acel.13129 (PMC7189995; doi:10.1111/acel.13129)
Supplement: Supplementary file 4 — Table S3 [file ACEL-19-e13129-s004.docx]

**Supplementary table 3**| Specific information about the survival curve.

| **Figure** | **Cell strain** | **Cell Number** | **Mean lifespan** | [**Increased**](javascript:;)  [**percent**](javascript:;) | ***p*-value** | **Type of microfluidic chip** |
| --- | --- | --- | --- | --- | --- | --- |
| Figure 1a | WT+AA | 96 | 21.01 | 50% | 8.36e-30(WT+SD) | U-shape chip |
|  | WT+GA | 91 | 18.62 | 33% | 1.25e-15(WT+SD) | U-shape chip |
|  | WT+Cur | 81 | 18.05 | 29% | 2.98e-12(WT+SD) | U-shape chip |
|  | WT+SD | 115 | 14.00 | - | - | U-shape chip |
| Figure 1b | WT+SD | 143 | 16.75 | - | - | U-shape chip |
|  | WT+EGCG | 138 | 22.93 | 64% | 1.31e-25(WT+SD) | U-shape chip |
| Figure 1e | WT+SD | 119 | 16.54 | - | - | U-shape chip |
|  | WT+NAC | 112 | 18.77 | 13% | 3.79e-5(WT+SD) | U-shape chip |
| Figure 2a | gcn5Δ | 97 | 18.40 | - | - | U-shape chip |
|  | gcn5Δ+EGCG | 83 | 16.05 | - | 3.92e-1(gcn5Δ) | U-shape chip |
|  | ngg1Δ | 83 | 16.25 | - | - | U-shape chip |
|  | ngg1Δ+EGCG | 80 | 15.15 | - | 5.38e-1(ngg1Δ) | U-shape chip |
| Figure 2b | hat1Δ | 110 | 13.27 | - | - | U-shape chip |
|  | hatΔ+EGCG | 110 | 19.03 | 43% | 2.29e-15(hat1Δ) | U-shape chip |
|  | hpa2Δ | 142 | 15.14 | - | - | U-shape chip |
|  | hpa2Δ+EGCG | 107 | 22.77 | 50% | 7.19e-16(hpa2Δ) | U-shape chip |
|  | rtt109Δ | 96 | 16.79 | - | - | U-shape chip |
|  | rtt109Δ+EGCG | 124 | 20.54 | 22% | 1.95e-11(rtt109Δ) | U-shape chip |
| Figure 2c | NGG1ngg1 | 80 | 23.85 | 39% | 1.70e-15(BY4743) | U-shape chip |
|  | GCN5gcn5 | 94 | 23.35 | 36% | 3.13-11(BY4743) | U-shape chip |
|  | BY4743 | 109 | 17.22 | - | - | U-shape chip |
| Figure 3a | ubp8Δ | 100 | 20.94 | - | - | island chip |
|  | ubp8Δ+EGCG | 100 | 25.26 | 21% | 3.80e-8(ubp8Δ) | island chip |
|  | sgf11Δ | 100 | 14.88 | - | - | island chip |
|  | sgf11Δ+EGCG | 100 | 24.74 | 66% | 3.13e-10(sgf11Δ) | island chip |
| Figure 3b | sus1Δ | 100 | 21.67 | - | - | island chip |
|  | sus1Δ+EGCG | 100 | 24.84 | 15% | 2.78e-4(sus1Δ) | island chip |
|  | sgf73Δ | 100 | 21.25 | - | - | island chip |
|  | sgf73Δ+EGCG | 100 | 24.82 | 17% | 2.27e-5(sgf73Δ) | island chip |
| Figure3c | sgf29Δ | 100 | 22.14 | - | - | island chip |
|  | sgf29Δ+EGCG | 100 | 24.98 | 13% | 2.54e-3(sgf29Δ) | island chip |
|  | ada2Δ | 100 | 20.50 | - | - | island chip |
|  | ada2Δ+EGCG | 100 | 25.04 | 22% | 7.03e-8(ada2Δ) | island chip |
| Figure 3f | K9A+SD | 124 | 24.34 | - | - | island chip+ U-shape chip |
|  | K9A+EGCG | 100 | 23.49 | - | 3.20e-1(K9A+SD) | island chip+ U-shape chip |
|  | K9R+SD | 98 | 25.18 | - | - | island chip+ U-shape chip |
|  | K9R+EGCG | 100 | 24.42 | - | 7.77e-1(K9R+SD) | island chip+ U-shape chip |
|  | K9Q+SD | 104 | 18.84 | - | - | island chip+ U-shape chip |
|  | K9Q+EGCG | 103 | 18.87 | - | 3.76e-1(K9Q+SD) | island chip+ U-shape chip |
| Figure 3g | K18A+SD | 111 | 21.22 | - | - | island chip+ U-shape chip |
|  | K18A+EGCG | 135 | 21.60 | - | 3.79e-1(K18A+SD) | island chip+ U-shape chip |
|  | K18R+SD | 118 | 21.58 | - | - | island chip+ U-shape chip |
|  | K18R+EGCG | 109 | 21.67 | - | 9.46e-1(K18R+SD) | island chip+ U-shape chip |
|  | K18Q+SD | 111 | 19.43 | - | - | island chip+ U-shape chip |
|  | K18Q+EGCG | 109 | 19.41 | - | 9.43e-1(K18Q+SD) | island chip+ U-shape chip |
| Figure 3h | K14A+SD | 80 | 15.92 | - | - | island chip+ U-shape chip |
|  | K14A+EGCG | 86 | 19.25 | 21% | 9.63e-5(K14A+SD) | island chip+ U-shape chip |
|  | K14R+SD | 77 | 18.50 | - | - | island chip+ U-shape chip |
|  | K14R+EGCG | 136 | 20.03 | 8% | 3.80e-3(K14R+SD) | island chip+ U-shape chip |
|  | K14Q+SD | 126 | 12.90 | - | - | island chip+ U-shape chip |
|  | K14Q+EGCG | 136 | 15.93 | 23% | 4.21e-6(K14Q+SD) | island chip+ U-shape chip |
| Figure 4a | BY4741-SD | 93 | 18.51 | - | - | island chip+ U-shape chip |
|  | BY4741-CR | 80 | 25.82 | 39% | 9.35e-24(BY4741-SD) | island chip+ U-shape chip |
|  | gcn5Δ -SD | 80 | 19.65 | - | - | island chip+ U-shape chip |
|  | gcn5Δ -CR | 80 | 19.62 | - | 7.06e-1(gcn5Δ -SD) | island chip+ U-shape chip |
|  | ngg1Δ-SD | 80 | 19.73 | - | - | island chip+ U-shape chip |
|  | ngg1Δ-CR | 80 | 19.10 | - | 1.54e-1(ngg1Δ-SD) | island chip+ U-shape chip |
| Figure 4d | BY4743-SD | 120 | 18.89 | - | - | island chip |
|  | BY4743-CR | 120 | 23.38 | 24% | 8.157299e-9(BY4743-SD) | island chip |
|  | GCN5gcn5-SD | 120 | 22.84 | - | - | island chip |
|  | GCN5gcn5-CR | 120 | 26.23 | 15% | 2.718064e-5(GCN5gcn5-SD) | island chip |
|  | NGG1ngg1-SD | 120 | 23.25 | - | - | island chip |
|  | NGG1ngg1-CR | 120 | 26.47 | 14% | 5.270451e-6 (NGG1ngg1-SD) | island chip |
| Figure 4e | K9A-SD | 130 | 22.78 | - | - | island chip+ U-shape chip |
|  | K9A-CR | 100 | 22.83 | - | 5.39e-1(K9A-SD) | island chip+ U-shape chip |
| Figure 4f | K14A -SD | 90 | 18.05 | - | - | island chip+ U-shape chip |
|  | K14A -CR | 100 | 23.97 | - | 6.03e-25(K14A -SD) | island chip+ U-shape chip |
| Figure 4g | K18A -SD | 81 | 19.72 | - | - | island chip+ U-shape chip |
|  | K18A -CR | 100 | 20.45 | - | 2.53e-1(K18A -SD) | island chip+ U-shape chip |
| Supplementary  figure 1 | fob1Δ+AA | 100 | 23.85 | - | 2.98e-3(fob1Δ+SD) | island chip |
|  | fob1Δ+GA | 100 | 24.93 | - | 4.85e-2(fob1Δ+SD) | island chip |
|  | fob1Δ+EGCG | 100 | 23.91 | - | 5.84e-3(fob1Δ+SD) | island chip |
|  | fob1Δ+SD | 100 | 27.20 | - | - | island chip |
