# Supplementary material for: Inhibition of histone acetyltransferase GCN5 extends lifespan in both yeast and human cell lines
Source: Aging Cell. 2020 Mar 11;19(4):e13129. doi: 10.1111/acel.13129 (PMC7189995; doi:10.1111/acel.13129)
Supplement: Supplementary file 5 — Table S4 [file ACEL-19-e13129-s005.docx]

| **Primer sequences used in qPCR** | |
| --- | --- |
| Saccharomyces cerevisiae | |
| Gene | Primer sequence（5’ to 3‘） |
| *actin* F | CACCATGTTCCCAGGTATTGC |
| *actin* R | TGGACCACTTTCGTCGTATTCT |
| *rtg3* F | TCCTGCCCAACAGCATGAAA |
| *rtg3* R | TCTACCGCCTTGGCTTGTTC |
| *msn4* F | TTGGCGACTTCTGGTGTTGA |
| *msn4* R | GTGTTTGGGCTTACCGTTGC |
| *gts1* F | GCAGCGACAACATCAACTCC |
| *gts1* R | TCGAAAACTGCTGGCTTTGC |
| *met32* F | ACCGGCAGAAAATGGTGTTG |
| *met32* R | TTGGGGTTTGCTTTTGCTGG |
| *stb4* F | GGAACTTGTCAGGAGGCCAT |
| *stb4* R | TGCTGCCAAAATGTCGCTTC |
| *hap1* F | TGAATGAAACGGGTGAGGCA |
| *hap1* R | CCCCCGTTTAGCTGGTTCAT |
| *met4* F | TTCCCGCTGACGCAACTAAT |
| *met4* R | TTCGGTGTGTGTGTTGTCCT |
| *dal80* F | AGTTTATGGTGAGCGGGTCC |
| *dal80* R | TTCGCAGGGGTCCAAAGAAA |
| *msn2* F | TCACCATTTCCCACAGCAGG |
| *msn2* R | TTCCTTCGTAACCCCAGCAC |
| *mot3* F | TGAACGTGGGCACCAATTCT |
| *mot3* R | AGTAGCGGTTGATTGAGCGT |
| *arr1* F | TGTGAAGAAAAACCGCCCAC |
| *arr1* R | CCAACACACACTGAGAGCAG |
| *ume6* F | TCTCAACCTCCAACTTCCGC |
| *ume6* R | CTTGACGAAGTGGCAAACGG |
| *gcn4* F | CTTACAACCGCAAACAGCGT |
| *gcn4* R | CTGGCGGCTTCAGTGTTTCT |
| Homo sapiens | |
| *gcn5* F | GCAAGGCCAATGAAACCTGTA |
| *gcn5* R | TCCAAGTGGGATACGTGGTCA |
| *ngg1* F | ACTGATGACCCTATCGACGTG |
| *ngg1* R | CCAGGGGTGGGATCTTGTAAT |
| *gapdh* F | ACCACAGTCCATGCCATCAC |
