# Supplementary material for: Inhibition of histone acetyltransferase GCN5 extends lifespan in both yeast and human cell lines
Source: Aging Cell. 2020 Mar 11;19(4):e13129. doi: 10.1111/acel.13129 (PMC7189995; doi:10.1111/acel.13129)
Supplement: Supplementary file 6 — Table S5 [file ACEL-19-e13129-s006.docx]

**Supplementary table 5**| **The information of yeast strains used.**

| **Strain Name** | **Genotype** |
| --- | --- |
| pJD47_H3_wild-type | MATa his3Δ200 leu2Δ0 lys2Δ0 trp1Δ63 ura3Δ0 met15Δ0 hht1-hhf1::NatMX4 can1::MFA1pr-HIS3 hht2-hhf2::URA3-HHTS-HHFS |
| Boeke-EMH-H3-9\|K9A | Same as pJD47_H3_wild-type, except hhts(H3K9A) |
| Boeke-EMH-H3-14\|K14A | Same as pJD47_H3_wild-type, except hhts(H3K14A) |
| Boeke-EMH-H3-23\|K23A | Same as pJD47_H3_wild-type, except hhts(H3K23A) |
| Boeke-EMH-H3-140\|K23R | Same as pJD47_H3_wild-type, except hhts(H3K23R) |
| Boeke-EMH-H3-154\|K14Q | Same as pJD47_H3_wild-type, except hhts(H3K14Q) |
| Boeke-EMH-H3-156\|K23Q | Same as pJD47_H3_wild-type, except hhts(H3K23Q) |
| YWD2000-WT | MATa his3-200 leu2∆1 ura3-52 trp1∆63 lys2-128 |
| YWD-H3K14A double | MATa his3-200 leu2∆1 ura3-52 trp1∆63 lys2-128 hht1(K14A) hht2(K14A) |
| YWD-H3K18A double | MATa his3-200 leu2∆1 ura3-52 trp1∆63 lys2-128 hht1(K18A) hht2(K18A) |
| YWD-H3K18R double | MATa his3-200 leu2∆1 ura3-52 trp1∆63 lys2-128 hht1(K18R) hht2(K18R) |
| YWD-H3K18Q double | MATa his3-200 leu2∆1 ura3-52 trp1∆63 lys2-128 hht1(K18Q) hht2(K18Q) |

Wild type and other single gene mutants from yeast mutant library from Krogan lab in ucsf.
